# Cotargeting of miR‐126‐3p and miR‐221‐3p inhibits PIK3R2 and PTEN, reducing lung cancer growth and metastasis by blocking AKT and CXCR4 signalling

**DOI:** 10.1002/1878-0261.13036

**Published:** 2021-07-21

**Authors:** Daniela Di Paolo, Francesca Pontis, Massimo Moro, Giovanni Centonze, Giulia Bertolini, Massimo Milione, Mavis Mensah, Miriam Segale, Ilaria Petraroia, Cristina Borzi, Paola Suatoni, Chiara Brignole, Patrizia Perri, Mirco Ponzoni, Ugo Pastorino, Gabriella Sozzi, Orazio Fortunato

**Affiliations:** ^1^ Laboratory of Experimental Therapies in Oncology IRCCS Istituto Giannina Gaslini Genoa Italy; ^2^ Tumor Genomics Unit Department of Research Fondazione IRCCS Istituto Nazionale dei Tumori Milan Italy; ^3^ First Pathology Division Department of Pathology and Laboratory Medicine Fondazione IRCCS Istituto Nazionale dei Tumori di Milano Italy; ^4^ Thoracic Surgery Unit Fondazione IRCCS Istituto Nazionale dei Tumori Milan Italy; ^5^ Present address: Nuclear Medicine Unit Santa Corona Hospital Pietra Ligure, Savona 17027 Italy

**Keywords:** lipid nanoparticles, lung cancer, microRNA

## Abstract

Lung cancer is the leading cause of cancer‐related death worldwide. Late diagnosis and metastatic dissemination contribute to its low survival rate. Since microRNA (miRNA) deregulation triggers lung carcinogenesis, miRNAs might represent an interesting therapeutic tool for lung cancer management. We identified seven miRNAs, including miR‐126‐3p and miR‐221‐3p, that are deregulated in tumours compared with normal tissues in a series of 38 non‐small‐cell lung cancer patients. A negative correlation between these two miRNAs was associated with poor patient survival. Concomitant miR‐126‐3p replacement and miR‐221‐3p inhibition, but not modulation of either miRNA alone, reduced lung cancer cell viability by inhibiting AKT signalling. PIK3R2 and PTEN were validated as direct targets of miR‐126‐3p and miR‐221‐3p, respectively. Simultaneous miRNA modulation reduced metastatic dissemination of lung cancer cells both *in vitro* and *in vivo* through CXCR4 inhibition. Systemic delivery of a combination of miR‐126‐3p mimic and miR‐221‐3p inhibitor encapsulated in lipid nanoparticles reduced lung cancer patient‐derived xenograft growth through blockade of the PIK3R2–AKT pathway. Our findings reveal that cotargeting miR‐126‐3p and miR‐221‐3p to hamper both tumour growth and metastasis could be a new therapeutic approach for lung cancer.

## Graphical abstract

Lung cancer is the first cause of cancer‐related deaths in the world. MicroRNAs are deregulated during lung carcinogenesis, and their modulation could represent an interesting therapeutic approach. We demonstrated that concomitant miR‐126 replacement and miR‐221 inhibition reduced lung cancer growth and metastasis both *in vitro* and *in vivo*. This study provides evidence for the use of miRNAs as therapy in lung cancer.

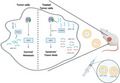

**Abbreviations**
CCL: coated cationic lipid nanoparticles
FACS: fluorescence‐activated cell sorter
HUVECs: human umbilical vein endothelial cells
ISH: *in situ* hybridization
LNA: locked nucleic acid
MILD: multicentric Italian lung detection
Mim: microRNA‐mimic
miRNA: microRNA
NHBE: normal human bronchial epithelial cells
NSCLC: non‐small‐cell lung cancer
OS: overall survival
PDX: patient-derived xenograft
S.E.M.: standard errors of the mean
SAECs: small airway epithelial cells
SCID: severe combined immunodeficient mice
siRNA: small interfering RNA
TRAIL: tumour necrosis factor-related apoptosis-inducing ligand
WB: western blot

## 1 Introduction

Lung cancer has the highest mortality rate among cancers, with a 5‐year survival rate of less than 18% [1]. Treatment options for lung cancer are influenced by the subtype and stage of disease. While surgery remains the gold standard in the treatment of lung cancer in patients with early‐stage disease [2], the combination of platinum‐based drugs is the standard of care for patients with advanced lung cancer [3]. Targeted therapies against epidermal growth factor and/or anaplastic lymphoma kinase have improved response rates only in a small group of patients with actionable mutations or rearrangements [4]. Moreover, over the last decade, the clinical management of lung cancer has been improved by the introduction of immune checkpoint inhibitors targeting cytotoxic T‐lymphocyte‐associated protein 4 and the programmed death 1/programmed death ligand 1 axis [5]. Unfortunately, only a small subset of patients successfully responds to this therapeutic approach as well [6], suggesting that finding alternative therapeutic strategies for lung cancer is an important clinical need.

MicroRNAs (miRNAs) are noncoding RNAs that are deregulated in different diseases, such as cancer, hepatitis and metabolic disease [7]. Based on their function, miRNAs may be considered ‘oncomirs’ when their overexpression in malignant cells inhibits tumour suppressor genes, for instance, the miR‐17‐92 cluster in lung cancer [8]. On the other hand, ‘tumour suppressor miRNAs’, such as let‐7a, are generally downregulated in cancer cells, and their loss results in aberrant expression of an oncogene [9]. miRNAs do not require perfect sequence complementarity with their target mRNAs to recognize them, implying that a single miRNA can regulate multiple genes [10] and several cellular mechanisms. This observation suggests a role for miRNAs as therapeutic tools in clinical cancer management [11].

Several studies have reported aberrant expression of miRNAs in lung tumours when compared to normal lung tissues [12], supporting the critical contribution of miRNAs to lung cancer development [13]. In our previous work, the miRNA expression profiles of 28 lung tumours and 24 paired normal lung tissues were analysed and the expression levels of the 34 identified miRNAs were revealed to be significantly different between normal lung and early‐stage lung cancer tissues [14]. Among the differentially expressed miRNAs, miR‐126‐3p was identified as an endothelial miRNA expressed at low levels in non‐small‐cell lung cancer (NSCLC) patients [15], and its replacement inhibited tumour growth by targeting EGFL7 [16]. Additionally, miR‐221 has been reported to be overexpressed in aggressive lung cancers, promoting tumour growth and invasion of lung cancer cells [17].

In the present study, miRNA expression was analysed in an independent clinical series of 38 lung cancer patients to identify the miRNAs whose expression modulation may have the greatest clinical utility. Here, we demonstrated that combined miR‐126‐3p replacement and miR‐221‐3p inhibition reduced tumour growth both *in vitro* and *in vivo* by inducing tumour necrosis factor‐related apoptosis‐inducing ligand (TRAIL)‐mediated apoptosis. Interestingly, miR‐126‐3p replacement reduced lung cancer metastatic dissemination through CXCR4 blockade both *in vitro* and *in vivo*. Importantly, the antitumour activity of these miRNAs encapsulated in lipid nanoparticles was also observed in patient‐derived xenograft (PDX) models of lung cancer.

## 2 Materials and methods

### 2.1 Population study

Tissue samples were collected from 38 lung cancer patients enrolled in the MILD trial (Table 1) [18]. Tissue specimens were obtained according to the Internal Review and the Ethics Boards of the Fondazione IRCCS Istituto Nazionale Tumori of Milan (INT 53/05), and the study was accomplished in accordance with the Declaration of Helsinki. All patients provided informed consent.

**Table 1:** Clinicopathological characteristics of patients for analysis on lung tissue.

	Trial MILD (*n* = 38 tissues)
Gender	
Male	30 (75%)
Female	10 (25%)
Age (years)	62.08 + 6 (S.D.)
Smoking habit (Pack‐Year index)	55.5 + 26.6 (S.D.)
Histotype	
Adenocarcinoma	27 (67.5%)
Squamous cell carcinoma	10 (25%)
Other	3 (7.5%)
Stage	
Ia–Ib	28 (70%)
II–III–IV	12 (30%)
Prognosis	
Alive	26 (65%)
Alive with disease	4 (10%)
Dead	8 (20%)
Not available	2 (5%)

### 2.2 Reagents and chemicals

Lipids are as follows: hydrogenated soy phosphatidylcholine (HSPC), cholesterol (CHE), 1,2‐distearoyl‐sn‐glycero‐3‐phosphoethanolamine‐*N*‐[methoxy(polyethylene glycol)‐2000] (DSPE‐PEG2000) and 1,2‐dioleoyl‐3‐trimethylammonium propane (DOTAP) (Avanti Polar Lipids, Inc., Alabaster, AL, USA). miRVana™ miRNA Mimic Custom miR‐126‐3p (m126) (ID# MC12841; Ambion, Thermo Fisher Scientific, Waltham, MA, USA) and miRCURY LNA™ miRNA Custom Inhibitor were used *in vivo* on a large scale [negative control A (SCR), ID# 339203YC10201876‐FZA; I‐HAS‐MIR221‐3p (i221), ID# 339203YC10202385‐FZA] (QIAGEN, Hilden, Germany). All other reagents were of analytical‐grade purity or the highest available purity (Sigma‐Aldrich, St. Louis, MO, USA).

### 2.3 Cell lines and siRNA, plasmid and miRNA transfection

The human lung cancer cell lines, Calu1, A549 and H460, were obtained from the American Type Culture Collection (ATCC, Manassas, VA, USA). Cells were cultured in RPMI 1640 (Gibco, Thermo Fisher Scientific, Waltham, MA, USA) medium supplemented with 10% heat‐inactivated FBS and 1% penicillin–streptomycin (Sigma‐Aldrich). Immortalized bronchial–epithelial cells and their genetically modified variants (HBEC1: hTERT + Cdk4; HBEC6: hTERT + Cdk4 + sh‐p53 + KRAS^V12^; HBEC‐KRAS: hTERT + Cdk4 + sh‐p53 + KRAS^V12high^) were obtained from J. Minna (UT Southwestern, TX) and were described previously [19]. Human primary normal human bronchial epithelial (NHBE) cells, small airway epithelial cells (SAECs) and human umbilical vein endothelial cells (HUVECs) were obtained from Lonza (Basel, Switzerland).

Cells were transfected with constructs at the following concentrations: negative control A mirVana miRNA mimic, negative control mim‐SCR, 50 nm (Ambion, Thermo Fisher Scientific) or miRCURY locked nucleic acid (LNA)‐SCR, 50 nm, (Exiqon, QIAGEN); mirVana miRNA mimic‐126‐3p (ID: MC12841; Ambion, Thermo Fisher Scientific), 50 nm; miRCURY miRNA inhibitor LNA‐221 (ID: YI04100607‐ADA, Exiqon, QIAGEN), 50 nm; and a combination of mimic‐126+LNA‐221 25 nm + 25 nm. Lipofectamine 2000 (Thermo Fisher Scientific) was used for transfections according to the manufacturer's instructions. PTEN transfection was performed using a PTEN expression plasmid (OriGene, Rockville, MD, USA) at 72 h following the company's protocol. PIK3R2 silencing was accomplished using 50 nm PI3K p85β small interfering RNA (siRNA; Santa Cruz, Dallas, TX, USA) according to the manufacturer's instructions after 72 h of transfection. CXCR4 silencing was performed using 100 nm MISSION^®^ esiRNA targeting human CXCR4 (EHU022821‐20UG; Sigma‐Aldrich) according to the manufacturer's instructions (72 h of transfection).

### 2.4 miRNA and gene expression analysis

RNA was extracted from cells at 72 h post‐transfection and from tissues using a mirVana Paris kit (Thermo Fisher Scientific). Reverse transcription and real‐time PCR were performed as previously described [20].

For gene expression analysis, cDNA synthesis was performed using 250 ng of total RNA. Relative quantification of the analysed genes was performed using a TaqMan assay (Thermo Fisher Scientific), and GAPDH was used as the endogenous control.

### 2.5 Viability assay

For the viability assay, cells were seeded into a 96‐well plate (5 × 10^3^ cells/well), and luminescence was measured after 72 h using a RealTime‐Glo MT Cell Viability Assay (Promega, Milan, Italy). Each experiment was performed in quintuplicate.

### 2.6 Apoptosis evaluation

Apoptosis was measured by quantifying the percentage of Annexin V/propidium iodide‐positive cells by flow cytometry (Miltenyi Biotech, Bergisch Gladbach, Germany) after 72 h of transfection, as previously described [20].

### 2.7 miRNA ISH

miRNA *in situ* hybridization (ISH) was performed on FFPE tissue sections as previously described [15]. Briefly, a combination of double DIG‐conjugated mirCURY LNA probes (Exiqon, Vedbæk, Denmark) and an automatic DAB chromogenic detection system was used for detection of miRNAs. Probes selected for ISH analysis are listed in Table S1. A scramble probe was used as negative control.

Samples were hybridized with probe mixture for 2 h, in the Dako Hybridizer, at specific probe hybridization temperature (RNA Tm −30 °C) (Table S1). After washing steps, miRNA expression was automated detected with the Ventana BenchMark ULTRA instrument using the OptiviewDAB Detection Kit (Ventana Medical Systems Arizona, Oro Valley, AZ, USA). For image analysis, stained sections were scanned with Aperio ScanScope XT (Leica Biosystems, Nussloch, Germany) and miRNAs signals were quantified as number of positive cells/total cells by counting three random field for each slide.

### 2.8 Proteomic array and western blot analyses

Proteins were extracted by incubation with RIPA buffer and quantified by the Bradford method. Forty‐five micrograms of protein was added to a human apoptosis array (R&D Systems, Minneapolis, MN, USA) and analysed following the manufacturer's instructions. Western blot (WB) analysis was performed as previously described [20] using the following antibodies: mouse anti‐cleaved caspase 8 (clone: 1C12; #9746), rabbit anti‐phospho‐AKT (Ser473) (clone: D9E; #4060), rabbit anti‐AKT (clone: 11E7; #4685), rabbit anti‐PIK3R2 (clone: 19H8; #4257), rabbit anti‐PTEN (#9552), rabbit anti‐TRAILR 1 (clone: D4E9; #8074), rabbit anti‐TRAILR 2 (clone: D9S1R; #42533), rabbit anti‐FADD (Human Specific; #2782), mouse anti‐FAS (clone: 4C3; #8023), rabbit anti‐p21( Waf1/Cip1; clone: 12D1 #2947), rabbit anticleaved caspase 3 (clone: Asp175; #9661) (all from Cell Signaling, Danvers, MA, USA, diluted 1 : 1000), rabbit anti‐CXCR4 (clone: UMB2; ab124824, Abcam, Cambridge, UK) mouse monoclonal anti‐β‐actin antibody (clone: AC‐74) (Sigma‐Aldrich A2228, 1 : 2000) horseradish peroxidase‐conjugated goat anti‐rabbit or goat anti‐mouse secondary antibodies (GE Healthcare, Chicago, IL, USA, 1 : 5000). Signal detection was performed via chemiluminescence (ECL, GE Healthcare) using a MINI HD9 Western Blot Imaging System (Cleaver Scientific Ltd, Warwickshire, UK). Band densities were quantified in imagej software (National Institutes of Health, Bethesda, MD, USA).

### 2.9 Flow cytometry

To analyse CXCR4 modulation, cells (A549, Calu1 and H460) were incubated with an anti‐human CD184 (CXCR4) monoclonal antibody, APC conjugated (clone: 12G5; # 17‐9999‐41 eBioscience, San Diego, CA, USA) diluted 1 : 100 in PBS for 15 min at room temperature and subsequently washed two times with PBS. Total events were analysed using a BD FACSCanto II with flowjo software (BD Biosciences, San Jose, CA, USA).

### 2.10 Migration, invasion and transendothelial migration assays

The migratory and invasive abilities of lung cancer cells (A549, Calu1 and H460) were assessed as previously described [20]. For the transendothelial migration assay, 1 × 10^5^ HUVECs were plated into the upper chambers of 24‐well cell culture inserts coated with Matrigel (BD Biosciences, Franklin Lakes, NJ, USA). One day later, 2 × 10^4^ PKH‐26‐labelled (A549, Calu1 and H460) cells were plated in the upper chambers of the inserts. For invasion, migration and transendothelial evaluation of four random fields per condition at 20× magnification were acquired after 48 h. The numbers of migrated cells (DAPI+ cells for invasion and migration assays and PKH‐26+ cells for transmigration assay) were counted using the imagej software.

### 2.11 Coated cationic lipid nanoparticles

The cationic lipid (DOTAP) amount is modified according to the number on negative charges of synthetic miRNA and miR‐LNA molecules [21, 22, 23]. Small, stable neutral coated cationic lipid nanoparticles (CCLs) encapsulating miRNA‐126 mimic (CCL‐126), miR‐221 LNA‐modified inhibitor (CCL‐221) or negative sequence (CCL‐SCR) molecules were prepared and purified as previously described [21, 22, 24]. The amounts and percentages of miRNAs encapsulated in the CCLs were evaluated by solubilizing lipid nanoparticle preparations with 40 mmol·L^−1^ sodium deoxycholate for 1.5 h at room temperature prior to spectrophotometric measurement at 260 nm. The good stability in media approximating the physiological conditions of the lipid nanoparticle preparations was evaluated by measuring the particle size, hydrophobic diameter, polydispersity index and zeta potential using a Malvern Nano ZS90 light scattering apparatus (Malvern Instruments Ltd, Worcestershire, UK).

### 2.12 IHC analysis

H&E staining was performed, and the levels of the Ki‐67, PTEN, PIK3R2 and cleaved caspase 3, cytokeratins and p‐AKT proteins were investigated using IHC staining. In brief, sections 2.5/3 μm thick were cut from paraffin blocks, dried, dewaxed, rehydrated and unmasked (with Dako PT‐link, EnVision™ FLEX Target Retrieval Solution, High/Low pH). Anti‐MIB1 (monoclonal; clone: MIB 1 Dako, Santa Clara, CA, USA, diluted 1 : 400), anti‐human cytokeratin (clones: AE1/AE3 – Dako – dilution 1 : 100; #M3515), anti‐PTEN (clone: A2B1: sc‐7974, diluted 1 : 50), (clone: 19H8; #4257; diluted 1 : 100), anti‐cleaved caspase 3 (clone: Asp175; #9661; diluted 1 : 100) and anti‐phospho‐AKT (Ser473) (clone: D9E; #4060; diluted 1 : 100), anti‐CXCR4 (clone: D4Z7W; #97680; diluted 1 : 800) (all from Cell Signaling) incubated with a commercially available detection kit (EnVision™ FLEX+, Dako) in an automated immunostainer (Dako Autostainer link 48). Images of sections were acquired with an Aperio ScanScope XT (Leica Biosystems, Aperio, Wetzlar, Germany) at 400× magnification, and four random fields were acquired for each condition. The percentage of positive cells was then evaluated by counting the positive cells out of the total number of cells. The Ki‐67 proliferation index was evaluated by the percentage of Ki‐67‐expressing cells among at least 500 cells counted in the areas of the strongest nuclear labelling (hot spots). For the necrosis evaluation, samples were stained with haematoxylin and eosin and the images were acquired with Aperio ScanScopeXT^®^ (Leica Biosystems, Aperio) at 20× and 200× magnifications.

The necrosis was quantified with the imagescope software (Aperio) using a positive pixel count algorithm. The staining intensity was classified as strongly positive (red), positive (orange), weakly positive (yellow) or negative (blue). The algorithm identifies yellow, orange and red as varying degrees of pink‐positive pixel of the eosin staining while blue as negative nuclei of deep blue‐purple colour‐pixel of haematoxylin staining. The percentage of necrosis in the tumour tissue samples, defined as increased eosinophilia in haematoxylin and eosin stains, was calculated as a total‐positive pixel staining divided by the total amount of pixels.

### 2.13 Cytokine quantification

The plasma levels of 12 cytokines (IL‐1α, IL‐1β, IL‐2, IL‐4, IL‐5, IL‐6, IL‐10, IL‐12, IL‐13, IL‐17a, G‐CSF and GM‐CSF) in mice treated with lipid nanoparticles for 4 weeks were analysed using Mouse Common Cytokines Multi‐Analyte ELISArray Kits following the manufacturer's instructions (Qiagen). The absorbance at 450 nm was measured in an Infinite M1000 plate reader (Tecan, GmbH, Grodig/Salzburg, Austria).

Interferon‐α and interferon‐β were analysed using Mouse IFN‐alpha and beta ELISA Kit (Abcam).

### 2.14 Luciferase assay

To investigate whether PIK3R2, PTEN and CXCR4 are a direct target of mir‐126 and miR‐221, the 3′UTR of PIK3R2 (ID: S813973), PTEN (ID: S809030) and CXCR4 (ID: S803938) were purchased from Switchgear Genomics and analysed as previously described [20]. Predicted target sites for miR‐126 and miR‐221 were mutated by direct mutagenesis of the pLightSwitch 3′UTR vectors encoding the 3′UTR of CXCR4, PTEN and PIK3R2, using the PCR‐based QuikChange II site‐directed mutagenesis kit (Agilent Technologies, Santa Clara, CA, USA) according to the manufacturer's instructions and the following primers:

CXCR4Mut‐FW 5′‐GTATGTCTCGTGTCCAGACTGTAGAAAAG‐3′
CXCR4Mut‐Rev 5′‐CTTTTCTACAGTCTGGACACGAGACATAC‐3′
PTENMut‐FW 5′‐AGGTTGTTCTACTGTGTCATGTATATAC‐3′
PTENMut‐Rev 5′‐GTATATACATGACACAGTAGAACAACCT‐3′
PIK3R2Mut‐FW 5′‐GCAGGTTTTGTATTTCACGTTGTTATTG‐3′
PIK3R2Mut‐Rev 5′‐CAATAACAACGTGAAATACAAAACCTGC‐3′

The presence of the mutations was confirmed by sequencing (Eurofins Genomics, Ebersberg, Germany).

### 2.15 PDXs and *in vivo* assays

Patient‐derived xenografts were established as previously described [25]. The PDX used for the experiment, derived from a lung cancer patient with a squamous cell carcinoma, stage IIIa. Mice were maintained in the Animal Facility of Fondazione IRCCS Istituto Nazionale dei Tumori. Animal experiments were authorized by the Institutional Animal Welfare Body and Italian Ministry of Health and were performed in accordance with national laws (D.lgs 26/2014). All experiments were carried out with female severe combined immunodeficient (SCID) mice aged 7–10 weeks (Charles River Laboratories, Calco, Italy). All animals were kept in IVC cages (max 5 animals/cage) in the Animal Facility of the ‘Fondazione IRCCS Istituto nazionale dei Tumori’. (SPF animal facility with 20 Hepa‐filtered air change/hour). Cages were maintained in rooms with controlled temperature and humidity, and 12/12‐light/dark cycles. Before starting experimentation, animals underwent at least 7 days of adaptation period. Cages were enriched with nesting material.

Lung cancer cells transfected (A549 and Calu1) with the miR‐126‐3p mimic, miR‐221‐3p inhibitor or SCR control were harvested 24 h post‐transfection and resuspended in Matrigel/RPMI (1 : 1). A total of 5 × 10^5^ cells were injected subcutaneously into both flanks of the mice (*n* = 5 tumours/group).

For the lung colonization assay, 5 × 10^5^ A549 or H460 cells transiently transfected with the miR‐126‐3p mimic or miR‐221‐3p inhibitor were injected intravenously into immunodeficient mice (*n* = 4 for each cell line). For the experimental metastasis assay, 3 weeks after injection, mice were sacrificed, and the lungs were harvested and analysed by both IHC staining and flow cytometry. Detection of tumour cells within dissociated murine lung tissue was performed by FACS analysis to exclude dead cells and MHC‐I‐positive murine cells, as previously reported [24].

Aerosol inhalation was performed by nebulizing 1.5 mg·kg^−1^ miR‐126‐3p in 200 µL of PBS in a 2‐chamber BIAS flow generator (model AIR421; EMMS Supporting Science, Edinburgh, UK). In brief, beginning 2 days after injection of H460 cells, mice were treated twice weekly for two weeks, and cell dissemination was analysed as described above.

For CXCR4 inhibition experiment, H460 cells (1 × 10^5^) in Matrigel (Corning, New York, NY, USA) + culture medium (1 : 1) were injected subcutaneously into the flanks of SCID mice. Mice were treated i.p. with 2 mg·kg^−1^ anti‐CXCR4 peptide R twice daily starting on the day after cell injection, and tumour growth was monitored by calliper measurement. At the end of the observation period (5 weeks), mice were sacrificed, and lungs were harvested for FACS and IHC analyses.

Mice bearing a PDX tumour in either flank were treated i.p. with CCL‐SCR, CCL‐126, CCL‐221 or an equimolar combination of CCL‐126 and CCL‐221 (CCL‐combo; total amount: 1.5 mg·kg^−1^) twice weekly for four weeks. Each time, mice received a total volume of 200 µL of a freshly diluted solution of lipid nanoparticles in HEPES‐buffered saline. The dose and schedule of treatment were selected based on our previous study [24]. Tumour growth was measured weekly using a calliper, and the results were analysed using graphpad prism 5 software (GraphPad Software, San Diego, CA, USA).

### 2.16 Statistical analysis

For the overall survival (OS) endpoint, the time‐to‐event occurrence was computed from the date of the surgery to the date when the event was recorded or the patient was censored at the date of the last follow‐up assessment (for event‐free patients). Survival curves were estimated using the Kaplan–Meier method and were compared by the log‐rank test. The Spearman correlation coefficient was also calculated. Statistical analyses were performed using graphpad prism 5 software. The results are presented as the mean ± standard errors of the mean (S.E.M.) values for quantitative data. Statistically significant differences were determined with Student's *t*‐test when comparing two groups or ANOVA test for multiple comparisons. For *in vivo* tumour growth, two‐way ANOVA test was used. *P*‐values < 0.05 were considered statistically significant.

## 3 Results

### 3.1 Analysis of miRNA levels in lung tumour tissues

**Fig. 1:**
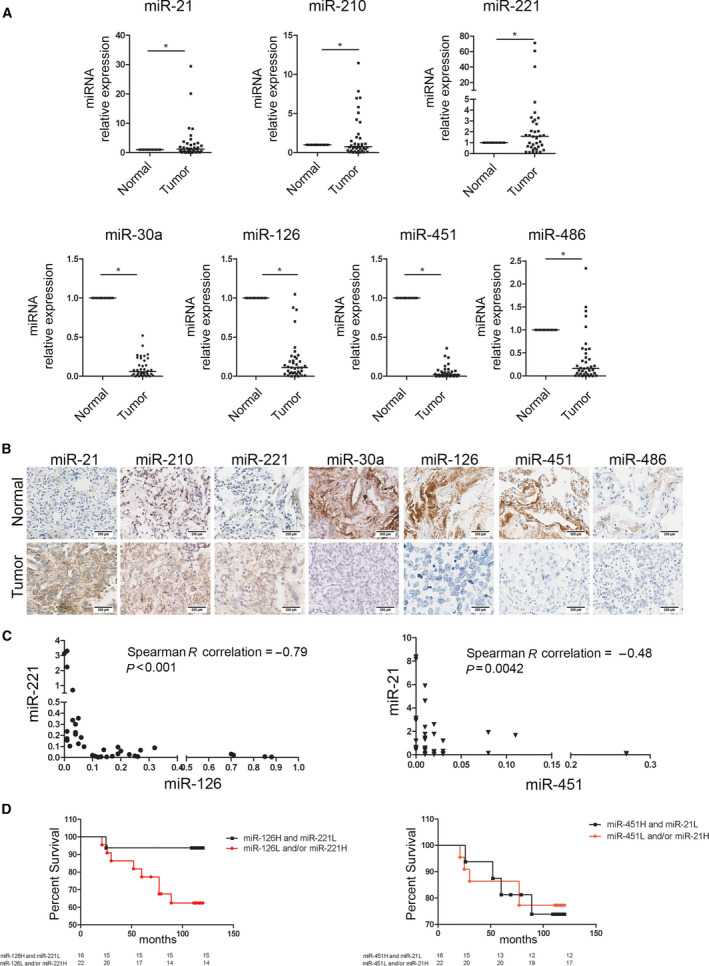
miRNAs deregulated in lung cancer tissues. (A) Dot plots showing miRNA median levels in lung cancers compared with distant normal tissues (*n* = 38 paired samples). *P < 0.05 versus controls. Data are expressed as mean ± standard error of the mean (S.E.M.). Statistically significant differences were determined with Student's *t*‐test. (B) miRNA levels in lung cancer tissues compared with normal tissues evaluated by miRNA ISH (*n* = 3). Scale bar: 200 µm. (C) Correlation graphs according to miR‐126/miR‐221 or miR‐451/miR‐21 expression levels in tumour tissues. (D) Kaplan–Meier curves illustrate the association with OS considering the presence of one or two unfavourable markers according to miRNA expression.

In our previous work [14], a microarray analysis of 28 tumours and 24 paired normal lung tissues from patients in a low‐dose computed tomography screening trial cohort was performed. In that study, 56 miRNAs were identified as differentially expressed between tumour and normal tissues. Among those miRNAs, 30 miRNAs with a potential role in lung carcinogenesis were identified by correlation analysis with the clinical characteristics of the patients. The association of miRNAs with disease aggressiveness was evaluated by RT‐PCR analysis of the expression of the previously identified miRNAs (Table S2) in 38 lung tumours and paired normal tissues from patients in the Multicentric Italian Lung Detection (MILD) screening trial [14]. Among the 30 analysed miRNAs, miR‐210, miR‐21 and miR‐221‐3p were overexpressed in lung tumours, whereas miR‐30a, miR‐126‐3p, miR‐451 and miR‐486 were downregulated in lung tumours compared with normal tissues (Fig. 1A). Furthermore, the deregulation of these miRNAs in tumour cells was confirmed and quantified by miRNA ISH in tissues from lung cancer patients (Fig. 1B and Fig. S1A).

Potential additive or synergistic effects of miRNAs deregulated in lung cancer tissues were assessed by correlation analysis between all the significantly modulated miRNAs to identify a more efficient anticancer strategy (Table S3).

Among all the differentially modulated miRNAs in lung cancer, we observed a significant negative correlation between miR‐126‐3p and miR‐221‐3p levels and between miR‐21 and miR‐451 levels, suggesting that these miRNAs could synergistically affect lung carcinogenesis (Fig. 1C). These findings led us to investigate the clinical utility of these miRNAs. Patient survival was evaluated according to the miRNA levels in tumour cells, with the median values of the investigated miRNAs used as the cut‐off values. As illustrated in Fig. 1D, 94% of patients with high miR‐126‐3p and low miR‐221‐3p levels in cancer tissues survived at 10 years, whereas the survival rate of patients with one or both unfavourable markers (miR‐126‐3p low and/or miR‐221‐3p high) was significantly lower 63% (Hazard Ratio 4.080, *P* = 0.0369) (Table S4). No differences were observed in the analysis of miR‐21 and miR‐451 levels according to patient survival (Fig. 1D and Table S5).

The expression of the identified miRNAs was confirmed in normal (NHBE, SAEC), nontumorigenic (HBEC1, HBEC6, HBEC‐KRAS^V12high^) and tumorigenic (H460, Calu1, A549) lung cell lines (Fig. S1B).

### 3.2 Concomitant modulation of miR‐126‐3p and miR‐221‐3p exhibits antitumour activity *in vitro*

To identify a potential therapeutic approach for lung cancer management, we developed a cotargeting strategy by simultaneously modulating miR‐126‐3p and miR‐221‐3p using commercially available synthetic oligonucleotides: a miR‐126‐3p mimic (m126) and a miR‐221‐3p inhibitor (i221). *In vitro* experiments were performed in A549 and Calu1 cells, which were selected because of the opposite miRNA levels detected in these cell lines (Fig. S1C,D).

**Fig. 2:**
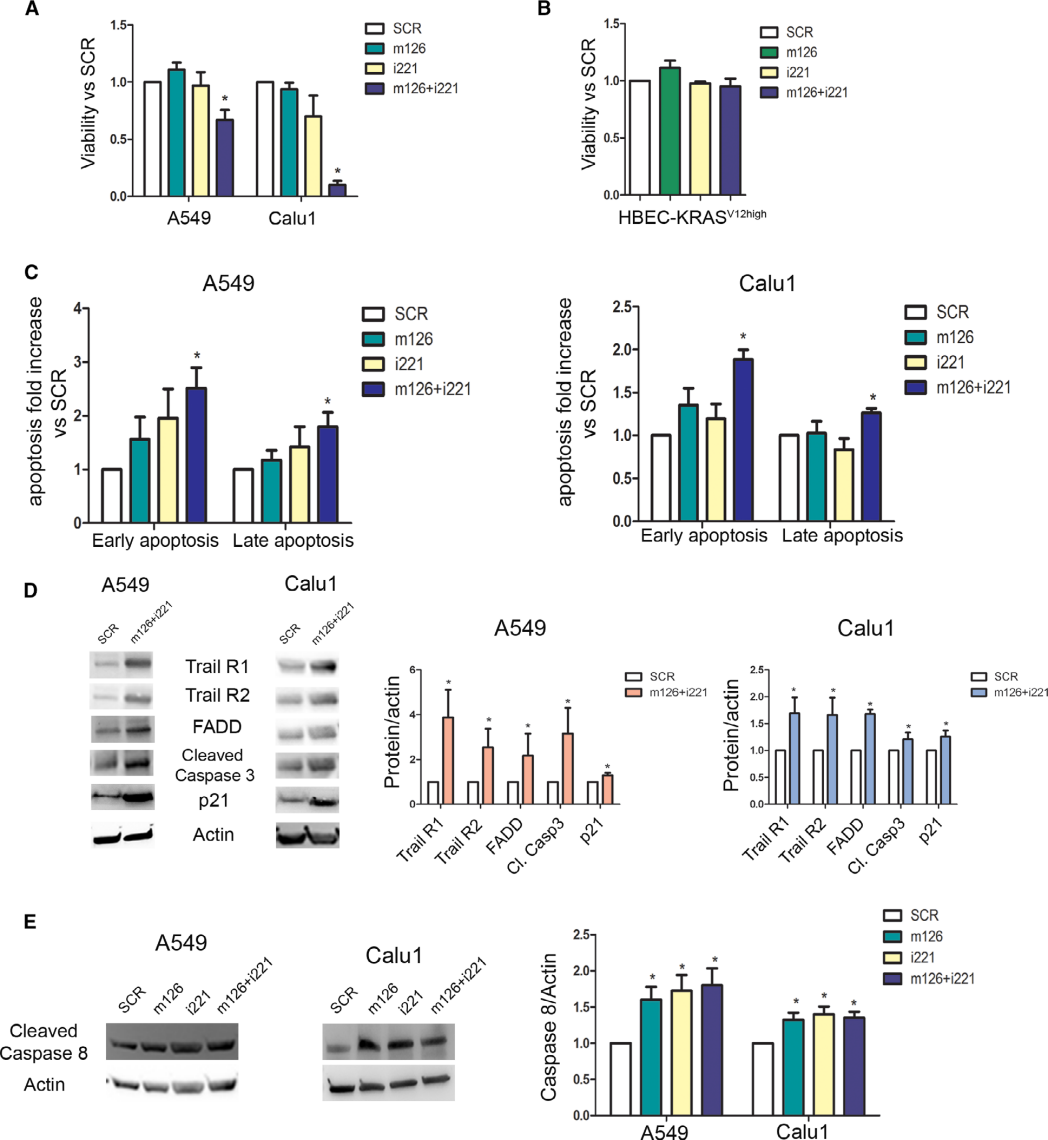
miR‐126‐3p and miR‐221‐3p modulation exhibited anti‐tumour activity *in vitro*. (A) Bar plots illustrate viability rate after miR‐126‐3p replacement and miR‐221‐3p inhibition compared with scramble (SCR) control (*n* = 5 for each cell). (B) Bar plots illustrate viability rate after miR‐126‐3p replacement and miR‐221‐3p inhibition compared with SCR control (*n* = 5 for each cell) in HBEC‐KRAS^V12high^ cells. (C) Flow cytometry analysis showed an increase in the number of early apoptotic (Annexin V+/PI−) and late apoptotic (Annexin V+/PI+) cells (*n* = 5 for each cell). (D) Representative images (left) and quantification (right) of western blot indicating the activation of apoptosis after miR‐126‐3p and miR‐221‐3p modulation (*n* = 3 for each cell). (E) Western blot band images (left) and quantification (right) demonstrated induction of caspase 8 after miRNA transfection (*n* = 5). Statistically significant differences were determined with Student's *t*‐test when comparing two groups or ANOVA test for multiple comparisons.*P < 0.05 versus SCR. Data are expressed as mean ± S.E.M.

First, we verified that the transfection with negative sequence for mimic (mim‐SCR) and for inhibitor (LNA‐SCR) did not alter proliferation of lung cancer cells as shown in Fig. S2A. Interestingly, compared with the negative sequence (SCR) control, only combined replacement of miR‐126‐3p with inhibition of miR‐221‐3p decreased the viability of all lung tumour cell lines after 72 h (Fig. 2A). Since single miRNA modulation did not affect proliferation, our results suggested a cooperative antitumour effect exerted by combined replacement of miR‐126‐3p and inhibition of miR‐221‐3p in lung cancer cells. Moreover, the antiproliferative effect of the combined treatment was also confirmed after 120 h post‐transfection (Fig. S2B). The proliferation rate of nontumorigenic human bronchial epithelial cells (HBEC‐KRAS^V12high^) was analysed to demonstrate whether miRNA modulation affects normal epithelial cells. As shown in Fig. 2B, we did not observe any significant changes in cell viability at 72 h after combined miR‐126‐3p replacement and miR‐221‐3p inhibition.

Moreover, the effects on apoptosis and the cell cycle were examined to better comprehend the means by which miRNA modulation caused the reduction in cancer cell viability. Only simultaneous modulation of both miRNAs resulted in increased numbers of early (Annexin V+/PI−) and late (Annexin V+/PI+) apoptotic cells at 72 h (Fig. 2C and Fig. S2C) and 120 h post‐transfection (Fig. S2E). However, we did not observe any significant changes in the cell cycle (Fig. S3A). We evaluated apoptosis in transfected HBEC‐KRAS^V12high^ cells and observed no changes in the numbers of apoptotic cells after 72 h compared with those in SCR control‐transfected cells (Fig. S3B). The activation of the apoptotic cascade was studied by analysis of a multiple protein array that detects 35 apoptosis‐related proteins. In all cell lines, simultaneous blockade of miR‐221‐3p and supplementation of miR‐126‐3p (m126+i221) increased the expression of TRAIL receptors 1 and 2 with concomitant upregulation of FADD and Fas, implying activation of the TRAIL‐dependent apoptotic pathway at 72 h post‐transfection (Fig. S3C). The activation of the apoptotic process was confirmed by the significant increase in the cleaved caspase 3 level in all m126+i221‐treated cells (Fig. S3D). Interestingly, the p21 level increased after modulation of miR‐126‐3p and miR‐221‐3p (Fig. S3D). The activation of the apoptotic cascade evidenced in Fig. S3C,D was also confirmed and quantified by WB analysis (Fig. 2D). An increase in the cleaved caspase 8 protein level after miRNA deregulation was observed in both cell lines (Fig. 2E), indicating the induction of TRAIL‐mediated apoptosis.

### 3.3 PIK3R2 and PTEN are direct targets of miR‐126‐3p and miR‐221‐3p

**Fig. 3:**
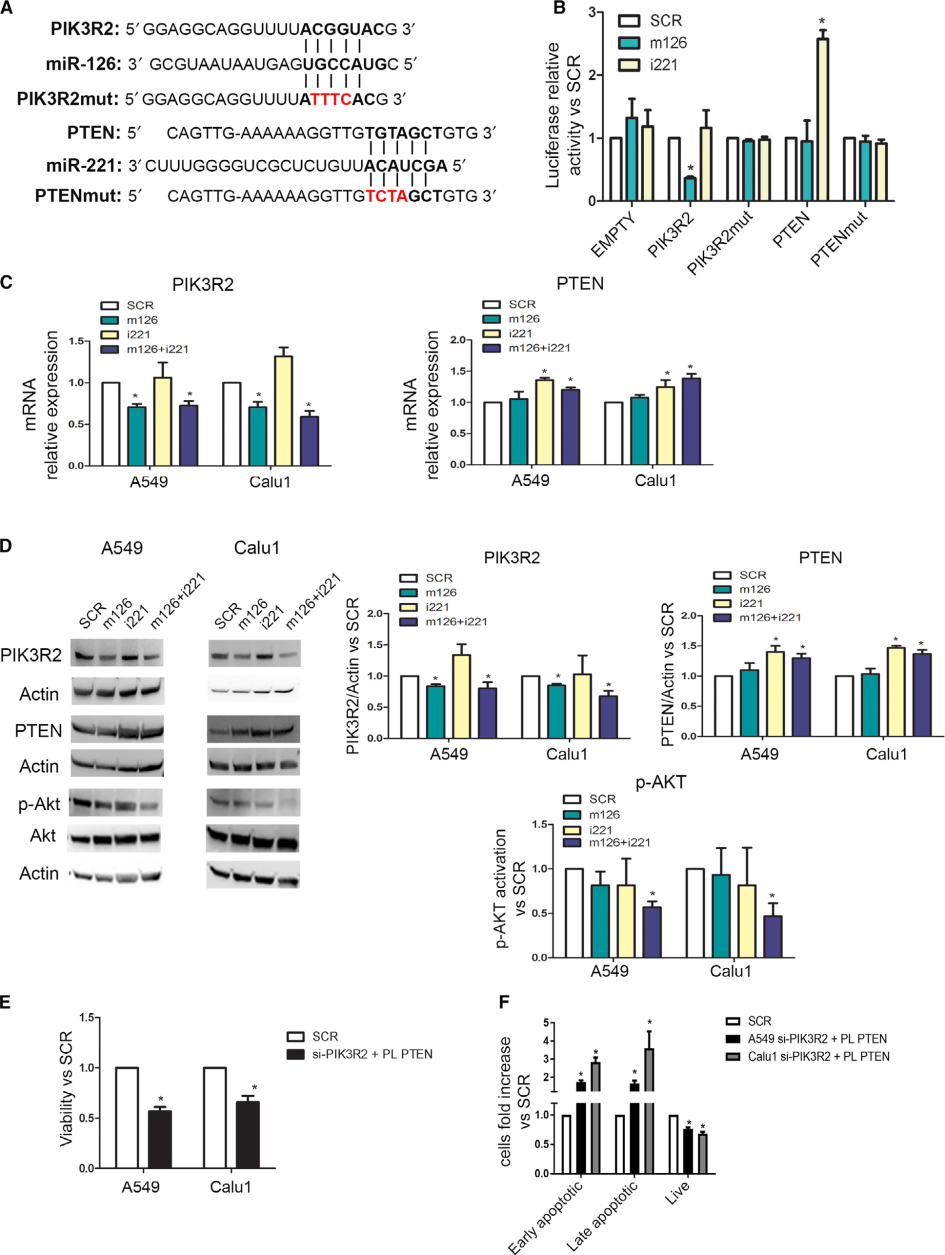
PIK3R2 and PTEN are direct targets of miR‐126‐3p and miR‐221‐3p. (A) miR‐126‐3p and miR‐221‐3p seed sequence alignments with the 3′UTRs of PIK3R2 and PTEN, respectively. (B) Bar graphs showing average luciferase activity of A549 transfected with PIK3R2, PTEN and EMPTY 3′UTR wild‐type or mutated in combination with miR‐126‐3p mimics or miR‐221‐3p inhibitors or control (*n* = 3). (C) Real‐time analysis of PIK3R2 and PTEN mRNA after miR‐126‐3p upregulation and miR‐221‐3p inhibition (*n* = 3 for each cell line). (D) Western blot bands and histogram revealed PIK3R2 downmodulation after miR‐126‐3p replacement. Inhibition of miR‐221‐3p increases PTEN expression as illustrated by western blot and bands' quantification (*n* = 3 for each cell). Phospo‐Akt (left) and histogram quantification (right) were observed after concomitant miRNA modulation (*n* = 3). (E) Silencing of PIK3R2 and overexpression of PTEN reduced viability of cancer cells (*n* = 5 for each cell). (F) The number of apoptotic cells was higher after silencing of PIK3R2 and PTEN upregulation (*n* = 5 for each cell). *P < 0.05 versus controls. Data are expressed as mean ± S.E.M.

Based on the observed *in vitro* results, putative targets of miR‐126‐3p and miR‐221‐3p, primarily those encoding proteins involved in the AKT pathway, were investigated by an *in silico* approach. This analysis revealed that the 3′UTRs of PIK3R2 and PTEN contain complementary binding sequences for miR‐126‐3p and miR‐221‐3p, respectively (Fig. 3A). The direct binding of these miRNAs with their targets was further demonstrated by a luciferase assay in A549 cells. Interestingly, we observed downmodulation of the luciferase activity of the PIK3R2 reporter after cotransfection with the miR‐126‐3p mimic and an increase in the PTEN reporter luciferase activity after cotransfection with miR‐221‐3p inhibitor (Fig. 3B). No modulation of luciferase activity was observed after cotransfection with an empty vector containing no 3′UTR or by site‐directed mutagenesis in the putative miRNA‐binding sites (Fig. 3B).

A significant reduction in PIK3R2 expression at either the mRNA (Fig. 3C) or protein (Fig. 3D) level was detected in all cell lines 72 h after miR‐126‐3p replacement alone or in combination with miR‐221‐3p inhibition. Furthermore, downmodulation of miR‐221‐3p expression in lung cancer cells increased the intracellular level of PTEN, as detected by qPCR (Fig. 3C) and western blotting (Fig. 3D). Importantly, only simultaneous miR‐126‐3p replacement and miR‐221‐3p inhibition resulted in blockade of AKT phosphorylation, as evaluated and quantified by WB (Fig. 3D) that may subtend the observed reduction in cancer cell viability (Fig. 2A).

To demonstrate that the antitumour effects of miR‐126‐3p and miR‐221‐3p were caused by the modulation of PIK3R2 and PTEN, PIK3R2 was silenced using a commercially available siRNA, and PTEN was overexpressed by transfection of a specific plasmid (Fig. S4A). After PIK3R2 silencing and PTEN overexpression, a reduction in cell viability compared with that in the control group was observed at 72 h post‐transfection (Fig. 3E). Moreover, an increase in the number of apoptotic cells (Fig. 3F) and concomitant blockade of AKT phosphorylation were observed in these cells after 72 h (Fig. S3A).

### 3.4 Concomitant modulation of miR‐126‐3p and miR‐221‐3p reduces lung metastatic dissemination

The potential role of the observed miRNAs in blockade of the lung cancer dissemination process was evaluated by analysing both the migratory and invasive abilities of three lung cancer cell lines (A549, Calu1 and H460). Based on our previous work [26], we analysed the anti‐metastatic activity of miR‐126 and miR‐221 also in the metastatic H460 lung cancer cells.

**Fig. 4:**
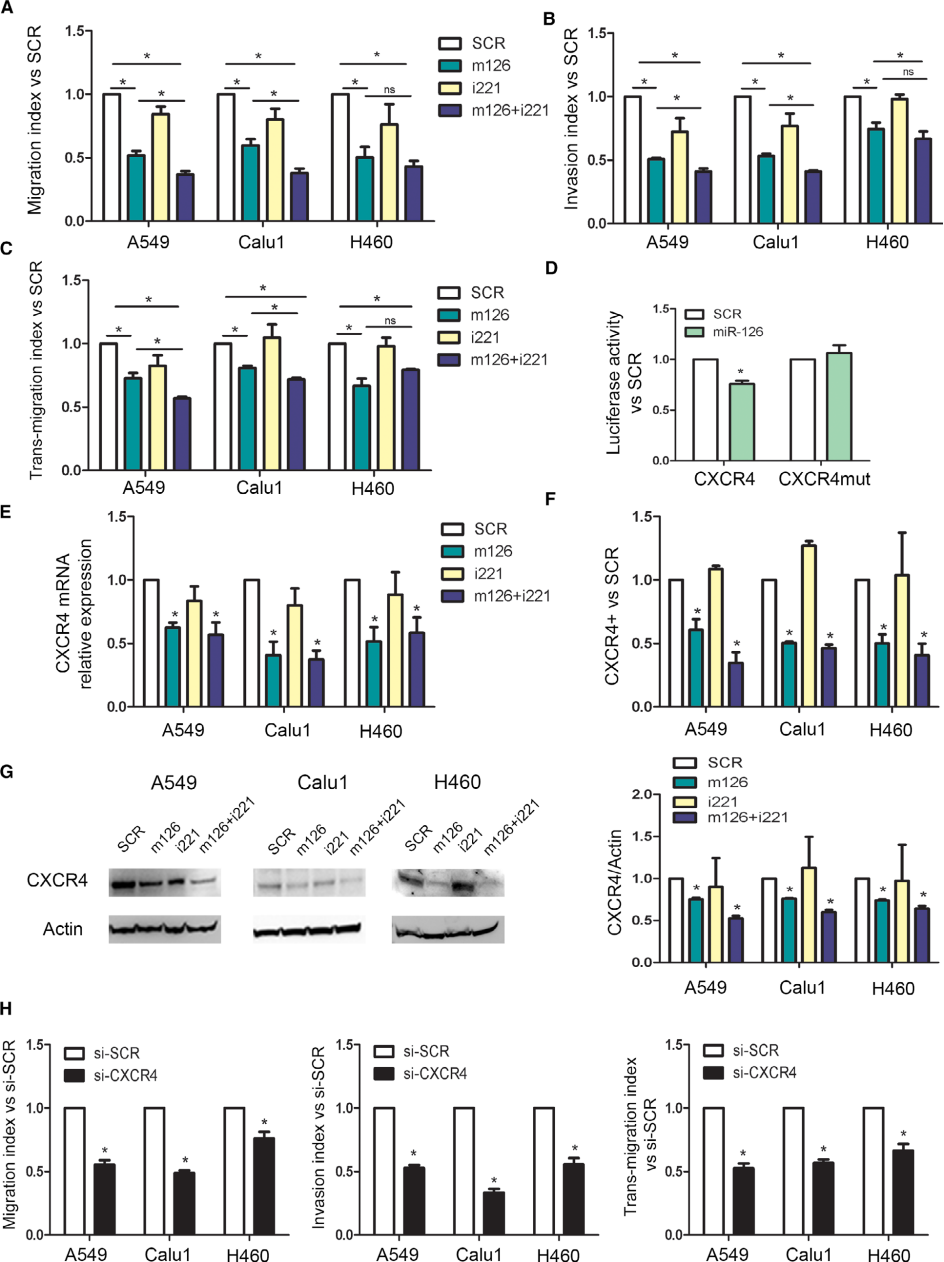
miR‐126‐3p replacement and miR‐221 inhibition reduced lung dissemination by inhibiting CXCR4 levels. (A) Migration index after miR‐126‐3p and miR‐221‐3p modulation of three lung cancer cells (*n* = 5). (B) Invasive capacity of lung cancer cells transfected with miR‐126/221 (*n* = 4). (C) Number of transmigrated lung cancer cells through endothelial monolayer after miRNA modulation (*n* = 3). (D) Luciferase activity for 3′UTR of CXCR4 wild‐type and mutated sequence after miR‐126 and SCR transfection in A549 cells (*n* = 4). CXCR4 levels after miR‐126‐3p replenishment and miR‐221‐3p inhibition evaluated by qPCR (*n* = 3) (E) and flow cytometry (*n* = 3) (F). (G) Representative images and quantification of CXCR4 in all the three cell lines after miRNA modulation (*n* = 3). (H) Migration, invasion and transendothelial index of lung cancer cells transient transfected with CXCR4 siRNA and controls (*n* = 3 for each cell line). Statistically significant differences were determined with Student's *t*‐test when comparing two groups or ANOVA test for multiple comparisons. *P < 0.05 versus controls. Data are expressed as mean ± S.E.M.

Interestingly, marked reductions in the cell migratory (Fig. 4A and Fig. S5A) and invasive (Fig. 4B and Fig. S5B) abilities after modulation of miR‐126‐3p alone or in combination with miR‐221‐3p were seen compared with those observed after transfection with the SCR control. Simultaneous miRNA modulation was more effective compared with miR‐126 alone, except for H460 cells likely due to the absence of miR‐221 (Fig. S1C). The *in vitro* transendothelial migration assay demonstrated that combination of miR‐126‐3p mimics and miR‐221 inhibitors reduced the number of cancer disseminating cells with respect to that of the corresponding control cells (Fig. 4C and Fig. S5C). To investigate whether the observed effect on migration is proliferation‐independent, we evaluated proliferation and demonstrated that it was not modulated by miRNA modulation after 24 h (Fig. S6A). Overall, these *in vitro* data indicate that combined miR‐126 replacement and miR‐221 inhibition are a candidate target that can inhibit the lung cancer dissemination process.

*In silico* analysis also revealed miR‐126‐3p binding sites in the 3′UTR of CXCR4, a well‐known receptor involved in metastatic dissemination of lung cancer cells [26]. We confirmed that miR‐126‐3p transfection in A549 caused significant reductions in luciferase activity in CXCR4 3′UTR whereas this inhibitory effect was absent after mutation in the binding site (Fig. 4D and Fig. S7A). miR‐126‐3p reduced CXCR4 mRNA (Fig. 4E) and protein expression using flow cytometry (Fig. 4F) and WB analysis (Fig. 4G). Inhibition of the CXCR4 pathway in transfected cells was also confirmed by the reduction in the phosphorylation of AKT, a downstream signalling molecule in the CXCR4 axis, compared with that in control cells (Fig. S7B).

Moreover, to better comprehend the key role of CXCR4 modulation in lung cancer dissemination, CXCR4 expression was silenced with a commercially available siRNA (si‐CXCR4) (Fig. S7C), and alterations in the migratory and invasive capacities were examined. Interestingly, si‐CXCR4 cells showed reduced migration, invasion and transendothelial migration compared with si‐SCR cells (Fig. 4H) that may be driven by reduction in AKT phosphorylation (Fig. S7C).

### 3.5 Combined miR‐126‐3p and miR‐221‐3p modulation reduces xenograft tumour growth in immunodeficient mice

**Fig. 5:**
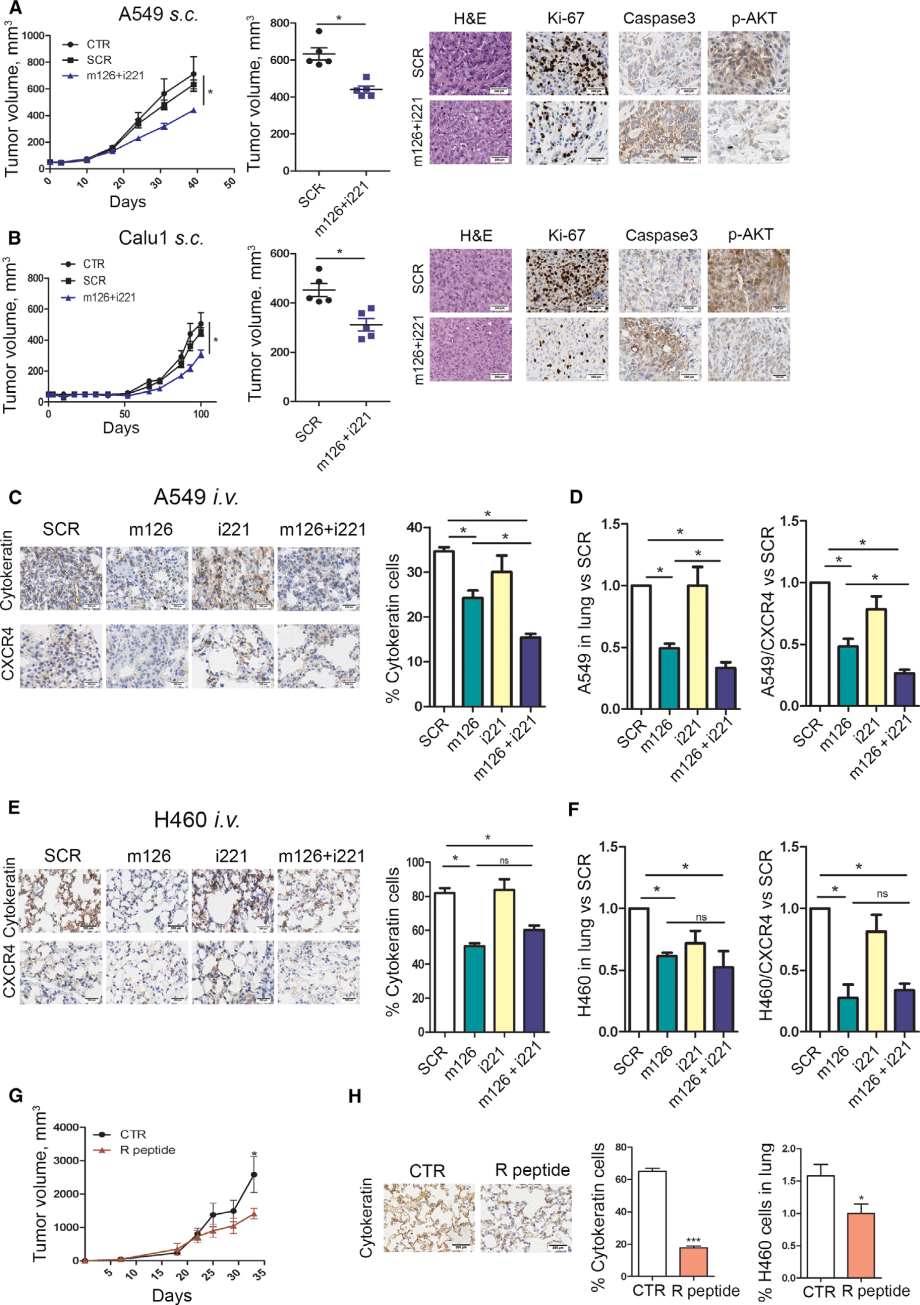
Combined replenishment of miR‐126‐3p and inhibition of miR‐221‐3p reduced xenograft growth *in vivo*. (A) A549 transfected with both miR‐126‐3p mimic (m126) and miR‐221‐3p inhibitor (i221) showed a reduction in tumour growth in immunodeficient mice compared with SCR and CTR controls. Ki‐67, cleaved caspase 3 and phosphor‐AKT quantification and representative images in A549 m126+i221 cells compared with controls. Necrosis was evaluated on haematoxylin & eosin (H&E) staining in subcutaneously implanted xenograft after miRNA transfection (*n* = 5 for each group) Scale bar: 200 µm (B) miR‐126‐3p replacement and miR‐221‐3p inhibition reduced tumour growth in subcutaneous Calu1 xenografts in immunodeficient mice. Ki‐67, cleaved caspase 3 and phospho‐AKT count and staining revealed a reduction in proliferation rate and increase in apoptosis of these cells. Necrosis was evaluated on H&E staining (*n* = 5 for each group). Scale bar: 200 µm. (C) Pan‐cytokeratin and CXCR4 staining and quantification in the lungs of mice treated with A549 transfected miR‐126 and/or miR‐221 alone or in combination (*n* = 4 for each group). Scale bar: 200 µm. (D) Quantification of A549 and A549/CXCR4+ cells in the lungs of mice by flow cytometry (*n* = 4). (E) Representative images and quantification of pan‐cytokeratin and CXCR4 staining of the lungs from H460 transfected with miR‐126‐3p, miR‐221 inhibitors alone or in combination and control (*n* = 4 mice for each group). Scale bar: 200 µm. (F) Flow cytometric quantification of H460‐disseminating cells and H460‐CXCR4 positive in the lung of treated mice (*n* = 4 mice for each group). (G) Tumour growth curves of mice treated with R peptide and CTR (*n* = 4 for each group). Scale bar: 200 µm. (H) Pan‐cytokeratin staining and flow cytometric quantification of the lungs from H460 treated with R peptide (*n* = 4 for each group). Data are expressed as mean ± S.E.M. Statistically significant differences were determined with Student's *t*‐test when comparing two groups or ANOVA test for multiple comparisons *P < 0.05, ***P < 0.0001.

Subcutaneous (s.c.) injection of A549 cells transiently transfected with both the miR‐126‐3p mimic and miR‐221 inhibitor into SCID mice resulted in a 30% reduction in tumour growth (Fig. 5A and Fig. S8A) compared to that in mice injected with SCR and nontransfected (CTR) control cells after 32 days, adding important *in vivo* functional validation. The antitumour efficacy of combined miR‐126‐3p and miR‐221‐3p modulation was supported by the decrease in the number of Ki‐67‐expressing proliferating cells in tumours generated from miR‐modulated cells compared with those generated from SCR‐treated cells and the increase in the necrotic area (Fig. S8A). miR‐126‐3p and miR‐221‐3p modulation exerted its antitumoral effect through the induction of apoptotic mechanisms in cancer cells, as shown by the increase in cleaved caspase 3‐positive cells and by blockade of AKT phosphorylation in treated tumours (Fig. 5A and Fig. S8A). A similar effect was also observed after subcutaneous injection of Calu1 cells transiently transfected with the combination of the miR‐126‐3p mimic and miR‐221‐3p inhibitor (Fig. 5B and Fig. S8B). Despite the similar size of tumours from Calu1 cells was observed, immunohistochemical (IHC) staining for Ki‐67 revealed a decrease in the mitotic index in m126+i221 Calu1 tumours compared with SCR Calu1 tumours and an increase in the necrotic area, as evaluated by IHC staining (Fig. S8B). Compared to SCR Calu1 tumours, m126+i221 Calu1 tumours also displayed an increase in the number of cleaved caspase 3‐positive cells and a downmodulation of phosphor‐AKT (Fig. 5B and Fig. S8B), overall suggesting that mir126+i221 treatment was able to induce apoptosis and antitumour effect also in this NSCLC cell line.

### 3.6 Combined miR‐126‐3p and miR‐221‐3p administration reduced lung cancer cell dissemination in mice

Since subcutaneous lung cancer xenografts do not disseminate efficiently to the lungs to form overt metastases [26], we performed an experimental metastasis assay in immunocompromised mice to show the efficacy of miRNA modulation in inhibiting A549 and H460 metastasis in two orthotopic models of lung cancer. Specifically, 5 × 10^5^ lung cancer cells transiently transfected with the miR‐126‐3p mimic and/or miR‐221 inhibitor or a control SCR sequence were injected into SCID mice via the tail vein, and lung nodule growth was monitored after cellular injection. First, we observed that combination of miR‐126 replacement and miR‐221 inhibition was able to reduce the growth of A549 cells in the lungs as shown by H&E and pan‐cytokeratin staining (Fig. 5C). Moreover, we observed a reduction in CXCR4‐positive cells compared with controls (Fig. 5C). Interestingly, also miR‐126 transfection alone was able to significantly reduce metastatic growth (Fig. 5C). Furthermore, by using an already validated gating strategy (Fig. S8C) [26], we observed a reduction in the human cells in the lungs of m126+i221 A549 cells compared with controls (Fig. 5D and Fig. S8D). H&E and pan‐cytokeratin staining demonstrated a reduction in the number of metastatic H460 cells in m126+i221 compared with control tumours (Fig. 5E and Fig. S8E). These data were also confirmed by flow cytometric analysis, which revealed a 40% reduction in the number of H460 cells in the lungs of mice injected with m126+i221 cells compared with that in the lungs of mice injected with control cells (Fig. 5F). Furthermore, replacement of miR‐126‐3p and miR‐221‐3p inhibition induced a reduction in the CXCR4 level in H460 cells in the lung 3 weeks postintravenous (i.v.) injection, as evaluated by determining CXCR4‐positive cells (Fig. 5F). No statistical difference was observed between m126+i221 H460 and m126 H460 in terms of metastasis reduction likely due to the absence of miR‐221 in this cell line.

To further investigate a new method for miRNA administration that could be potential used for clinical applications, we performed a preliminary experiment by treating the lungs via nebulized aerosol inhalation. So to further confirm the importance of miR‐126‐3p in inhibiting metastatic dissemination of lung cancer cells, we injected H460 cells into SCID mice via the tail vein, and after 1 week, mice were treated twice with naked miR‐126‐3p mimics (*n* = 4 for each group). After confirmation of miR‐126‐3p replacement in the lungs (Fig. S9A), we observed a reduction in H460 cells compared with that in mice treated with the SCR control, as evaluated by IHC staining (Fig. S9B) and flow cytometry (Fig. S9C). Interestingly, miR‐126‐3p mimic treatment reduced the number of CXCR4‐positive H460 cells in the lungs compared with that in mice treated with the SCR control (Fig. S9D).

A cyclic peptide inhibitor of CXCR4 (peptide R), designed as an SDF‐1 mimetic peptide, and already validated to inhibit CXCR4 downstream pathways and to exert anti‐metastatic effects, was utilized to better explain the role of CXCR4 inhibition in the modulation of lung cancer metastatic potential [27, 28]. In detail, H460 cells were inoculated subcutaneously into SCID mice, and the mice were treated daily with peptide R (2 mg·kg^−1^). We observed a slight reduction in subcutaneous tumour growth after 32 days (Fig. 5G) but a consistent reduction in the dissemination of H460 cells to the lungs (a reduction of 60% compared with CTR), as evaluated by both IHC staining and flow cytometry (Fig. 5H).

### 3.7 CCL‐combo administration reduces tumour growth in PDX models

**Fig. 6:**
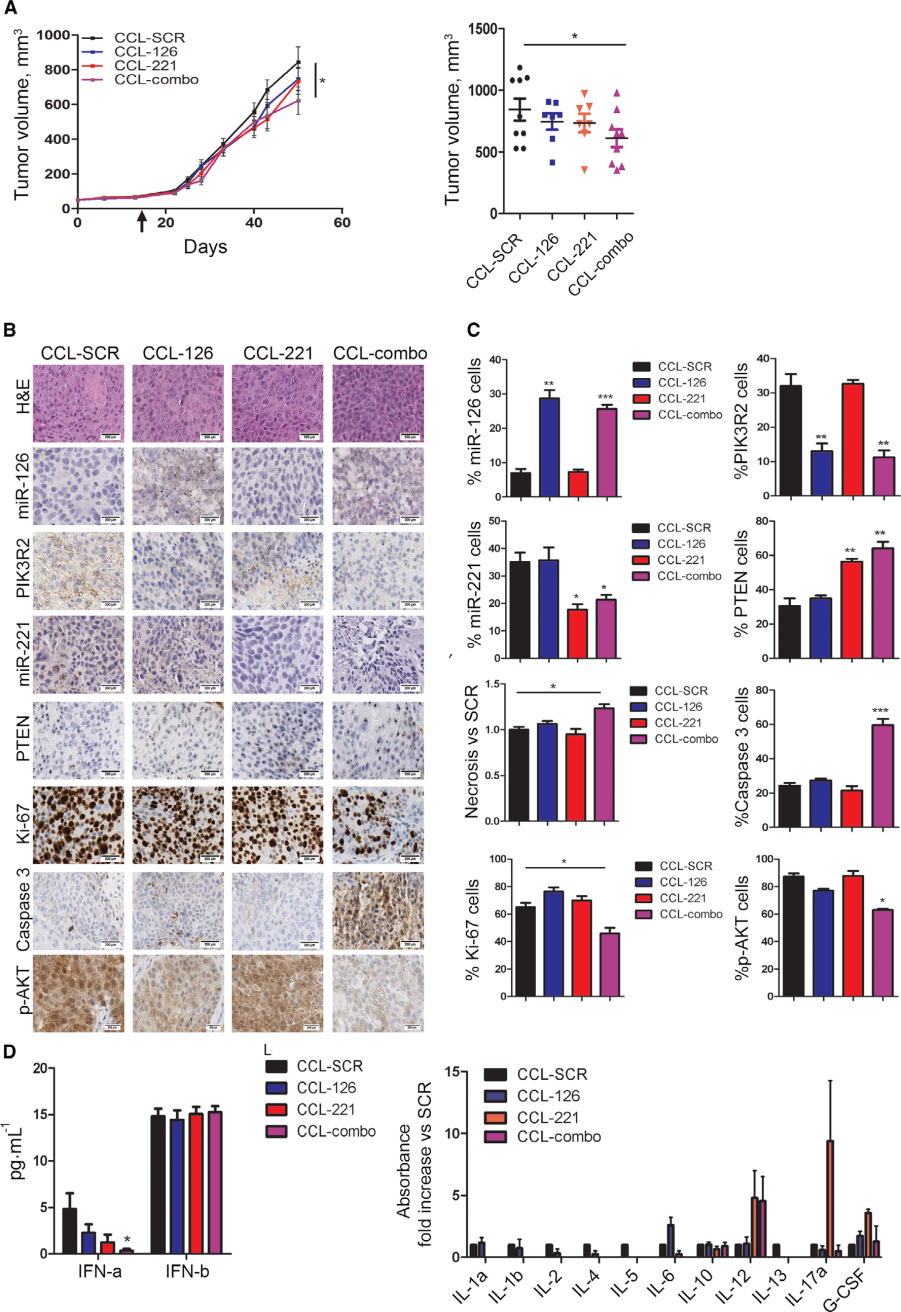
Combined replenishment of miR‐126‐3p and inhibition of miR‐221‐3p reduced tumour growth *in vivo*. (A) Tumour growth curves and tumour volumes of PDX bearing mice, treated with 1.5 mg·kg^−1^ of negative miRNA inhibitor sequence (SCR) (*n* = 9) or a equimolar miR‐126‐3p mimic or miR‐221‐3p inhibitor sequence alone (*n* = 7) or in combination (combo) (*n* = 9) entrapped in neutral lipid nanoparticles (CCL), two times a week for four weeks. (B) Immunohistochemistry (IHC) images and graphs (C) show the proliferation index, miR‐126‐3p, miR‐221‐3p, necrosis and PTEN, PIK3R2 and CXCR4 protein levels at the end of treatment compared with those in control mice (*n* = 7). Scale bar: 200 µm. (D) Bar graphs illustrated plasma levels of interferon (IFN)‐α and β in the CCL‐treated mice (left) (*n* = 5). Graphs illustrated 12 plasma pro‐inflammatory cytokine levels in the CCL‐combo‐treated mice twice after 4 weeks of treatment (right) (*n* = 3). Data are presented as means ± S.E.M. *P < 0.05 versus SCR, **P < 0.01, ***P < 0.001. Black arrow indicates the starting of the treatment.

The efficacy of combined modulation of miR‐126‐3p and miR‐221‐3p as a novel therapeutic approach for lung cancer management was evaluated by using neutral CCLs [24, 29] encapsulating miR‐126‐3p mimic and miR‐221‐3p inhibitor molecules administered alone (CCL‐126 and CCL‐221, respectively) or in combination (CCL‐combo). In the PDX model, mice were treated intraperitoneally (i.p.) twice weekly for four weeks with CCLs. Figure 6A shows the clear reduction (33%) in the volume of CCL‐combo‐treated PDXs compared to PDXs treated with CCLs containing a negative sequence (CCL‐SCR). PDXs treated with CCL‐126 or CCL‐221 alone did not show a significant reduction in tumour growth compared to those treated with CCL‐SCR. Effective CCL delivery was confirmed by ISH (Fig. 6B,C). The miR‐126/miR‐221 combination also decreased the number of Ki‐67‐positive proliferating cells and also AKT phosphorylation in PDXs (Fig. 6B,C). The reduction in tumour proliferation was confirmed by the 20% increase in the necrotic area observed only in CCL‐combo‐treated tumours and not in tumours treated with CCL‐SCR or CCL‐126/221 alone (Fig. 6B,C). Interestingly, CCL‐combo treatment reduced tumour growth by activating the apoptotic cascade, as demonstrated by the increase in caspase 3‐positive cells (Fig. 6B,C). IHC staining demonstrated the increase in PTEN and downmodulation of PIK3R2 in CCL‐combo treated PDXs (Fig. 6B,C). The modulation of miRNAs and their direct targets PIK3R2 and PTEN was confirmed by real‐time PCR (Fig. S10B,C). Interestingly, cell cycle arrest was also demonstrated by the increase in p21 mRNA expression in CCL‐combo‐treated tumours (Fig. S10C). The safety of our lipid nanoparticles was previously demonstrated *in vivo* [24, 29]. To exclude nonspecific interferon induction after lipid nanoparticle treatment, we evaluated the levels of interferon alpha (IFN‐α) and beta (IFN‐β) at the end of CCL treatment. Compared to CCL‐SCR, CCL‐combo reduced the IFN‐α expression whereas and CCL‐126 and CCL‐221 alone did not modulate the plasma levels of IFN‐α and β in treated mice (Fig. 6D). Additionally, a panel of 12 pro‐inflammatory cytokines was evaluated to exclude potential chronic toxic effects of the lipid nanoparticles. No appreciable changes in the plasma levels of these cytokines, except for a small increase in IL‐12, were observed in mice treated with CCL‐combo or CCL‐SCR. Therefore, immunological responses to the carrier can be excluded (Fig. 6D). Collectively, these data confirmed the importance of combined treatment with a miR‐126‐3p mimic and a miR‐221‐3p inhibitor to reduce lung cancer growth *in vivo*.

## 4 Conclusion

Several works have elucidated the importance of miRNAs as crucial modulators of cancer development [10]. In particular, we and others have demonstrated that several miRNAs are deregulated in lung cancer tissues and are responsible for the abnormal phenotype of cancer cells [20, 30]. In the current study, upregulation of miR‐210, miR‐21 and miR‐221/222, together with downmodulation of miR‐126‐3p, miR‐486, miR‐451 and miR‐30a, was validated in an independent series of early‐stage (70% stage I) lung cancer patients enrolled in the MILD lung cancer screening trial.

In addition to the previously reported antitumour effects of modulating single miRNAs in cancer cells, our findings here highlighted that combined modulation of two miRNAs has to be envisaged to increase antiproliferative and anti‐metastatic dissemination effects in lung cancer both *in vitro* and *in vivo*.

Downregulation of miRNA‐126 has been described in different malignancies such as colorectal, liver, breast and renal cancers [31, 32] and in lung tissues [15, 16, 33, 34]. This downregulation was also confirmed in the current study, revealing this miRNA as a tumour suppressor miRNA. Interestingly, a study by Song and colleagues showed that miR‐126 replacement reduced the proliferative, migratory and invasive abilities of only one lung cancer cell line [34]. In our previous study, H460 lung cancer cells injected intravenously into SCID mice invaded the lung parenchyma and produced fully developed metastatic nodules [26]. In a similar lung colonization assay, miR‐126 reduced the ability of tumour cells to colonize the lung parenchyma and generate metastases, suggesting that this miRNA plays a role in metastatic processes. Indeed, simultaneous miR‐126 replacement and miR‐221 inhibition reduced the migratory and invasive capacities of lung cancer cells through blockade of the CXCR4 axis. The CXCR4 axis plays a pivotal role in the regulation of cancer cell dissemination and colonization of distant organs [35, 36]. CXCR4/SDF‐1 axis targeting has been demonstrated in several preclinical and clinical studies as a promising therapeutic strategy for metastatic disease in different cancers, including NSCLC [35, 37, 38]. The anti‐metastatic potential of combined miR‐126 and miR‐221 modulation was further demonstrated by an *in vivo* colonization assay using two different lung cancer cells.

An important overexpressed miRNA cluster in lung cancer is miR‐221/222, which has previously been described to be upregulated in breast cancer, colorectal cancer and glioblastoma [39]. miR‐221 has been reported to play a role in lung cancer metastasis aggressiveness [40] and to be associated with the poor prognosis of NSCLC patients [41]. A previous study demonstrated that miR‐221 overexpression induced TRAIL activation [42]. In particular, transfection with miR‐221/222 inhibitors rendered lung cancer cells sensitive to TRAIL through p27. Interestingly, our data revealed that inhibition of miR‐221‐3p induced apoptosis by activating TRAIL in the two studied lung cancer cell lines. Importantly, we showed that downmodulation of miR‐221‐3p increased PTEN protein expression, leading to a reduction in AKT phosphorylation with a negative effect on cell viability, only in combination with miR‐126‐3p replacement. The potential therapeutic use of miRNA modulators in lung cancer is based on the rationale that miRNAs can revert malignant phenotypes and aggressiveness [43]. Our study demonstrated that only combined miR‐126‐3p restoration and miR‐221‐3p inhibition reduced tumour growth by simultaneously inhibiting the PI3 kinase (PI3K)/AKT/PTEN pathway in PDX models. We observed that combined miR‐126‐3p and miR‐221‐3p modulation is well tolerated in normal cells with adequate levels of endogenously expressed miRNAs, while in tumour cells, it restores a balance that reactivates physiological mechanisms of gene/pathway regulation, leading to antitumour effects.

Here, we established a proof of concept for a new therapeutic strategy based on combined administration of lipid nanoparticles containing a miR‐126‐3p mimic and a miR‐221‐3p inhibitor. These nanoparticles showed the ability to reduce the growth of lung cancer PDXs in immunodeficient mice. The observed mild reduction in tumour growth was probably due to the low delivery efficiency of the untargeted lipid nanoparticles, as reported in our previous work [24]. To optimize the antitumour efficacy of our lipid nanoparticles, careful selection and addition of lung cancer‐specific ligands to the lipid surface could result in an improvement essential for the therapeutic use of these compounds in lung cancer. Furthermore, since miRNAs have shown slight but significant therapeutic potential in cancers, future strategies combining neutral lipid nanoparticles encapsulating synthetic miRNAs with standard chemotherapeutic agents could improve the efficacy of the antitumour activity of these novel drugs. Despite challenges such as the development of novel delivery carriers or the onset of chemoresistance, the combination of miRNAs and chemotherapeutic agents constitutes an interesting approach for lung cancer treatment.

Our study reports a potential new therapeutic approach for lung cancer management that simultaneously targets two deregulated miRNAs in lung cancer tissues. Interestingly, only when miR‐126‐3p and miR‐221‐3p were modulated simultaneously was antitumour activity observed, in contrast to when each miRNA was modulated alone, suggesting that combined modulation of miRNAs could be a new approach for anticancer therapy.

## Conflict of interest

The authors declare no conflict of interest.

## Author contributions

DDP, FP, MM, OF, GB designed the study, acquired, analysed and drafted the article; FP, GC, MMe, MS, CB, PS, CBr, IP acquired and analysed data; MMi, PP, MP, UP, GS designed the study, interpreted data, revised critically the article. All authors read and approved the final manuscript.

### Peer Review

The peer review history for this article is available at https://publons.com/publon/10.1002/1878‐0261.13036.

## Supporting information


**Fig. S1.** miRNAs de‐regulation in lung cancer.
**Fig. S2.** Proliferation and apoptosis analysis.
**Fig. S3.** Apoptosis analysis.
**Fig. S4.** PIK3R2 and PTEN are miR‐126 and miR‐221 targets.
**Fig. S5.** Migratory and invasive capacity of lung cancer cells.
**Fig. S6.** Proliferation analysis after 24 h post transfection.
**Fig. S7.** CXCR4 is fundamental for metastatic dissemination.
**Fig. S8.** In vivo assays.
**Fig. S9.** miR‐126 replacement using nebulized aerosol inhalation.
**Fig. S10.** PDX was treated with lipid‐nanoparticles.

**Table S1.** List of detection probes.
**Table S2.** List of miRNAs analyzed in lung cancer tissues.
**Table S3.** Correlation analysis between de‐regulated miRNAs in lung cancer tissues.
**Table S4.** Contingency table of lung cancer patients.
**Table S5.** Contingency table of lung cancer patients.

## Data Availability

The data that support the findings of this study are available from the corresponding author orazio.fortunato@istitutotumori.mi.it upon reasonable request.
