# Supplementary material for: Cotargeting of miR‐126‐3p and miR‐221‐3p inhibits PIK3R2 and PTEN, reducing lung cancer growth and metastasis by blocking AKT and CXCR4 signalling
Source: Mol Oncol. 2021 Jul 21;15(11):2969–88. doi: 10.1002/1878-0261.13036 (PMC8564655; doi:10.1002/1878-0261.13036)
Supplement: Supplementary file 1 — Fig. S1. miRNAs de‐regulation in lung cancer. Fig. S2. Proliferation and apoptosis analysis. Fig. S3. Apoptosis analysis. Fig. S4. PIK3R2 and PTEN are miR‐126 and miR‐221 targets. Fig. S5. Migratory and invasive capacity of lung cancer cells. Fig. S6. Proliferation analysis after 24 h post transfection. Fig. S7. CXCR4 is fundamental for metastatic dissemination. Fig. S8. In vivo assays. Fig. S9. miR‐126 replacement using nebulized aerosol inhalation. Fig. S10. PDX was treated with lipid‐nanoparticles. [file MOL2-15-2969-s001.pdf]

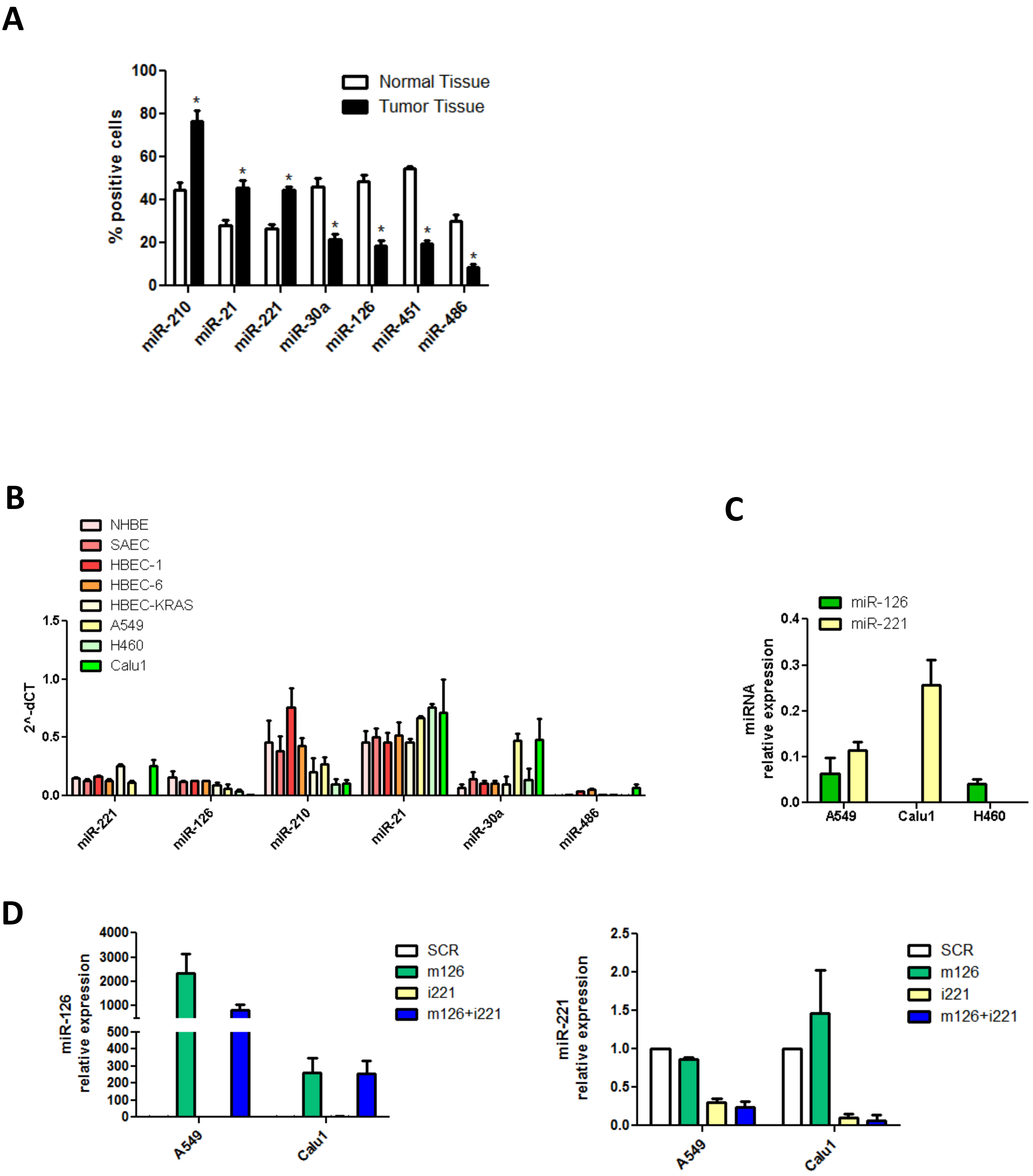
A
% positive cells
Normal Tissue
Tumor Tissue
miR-210
miR-21
miR-221
miR-30a
miR-126
miR-451
miR-486
B
NHBE
SAEC
HBEC-1
HBEC-6
HBEC-KRAS
A549
H460
Calu1
2^-dCT
C
miR-126
miR-221
miRNA relative expression
A549
Calu1
H460
D
miR-126 relative expression
SCR
m126
i221
m126+i221
miR-221 relative expression


Figure S1

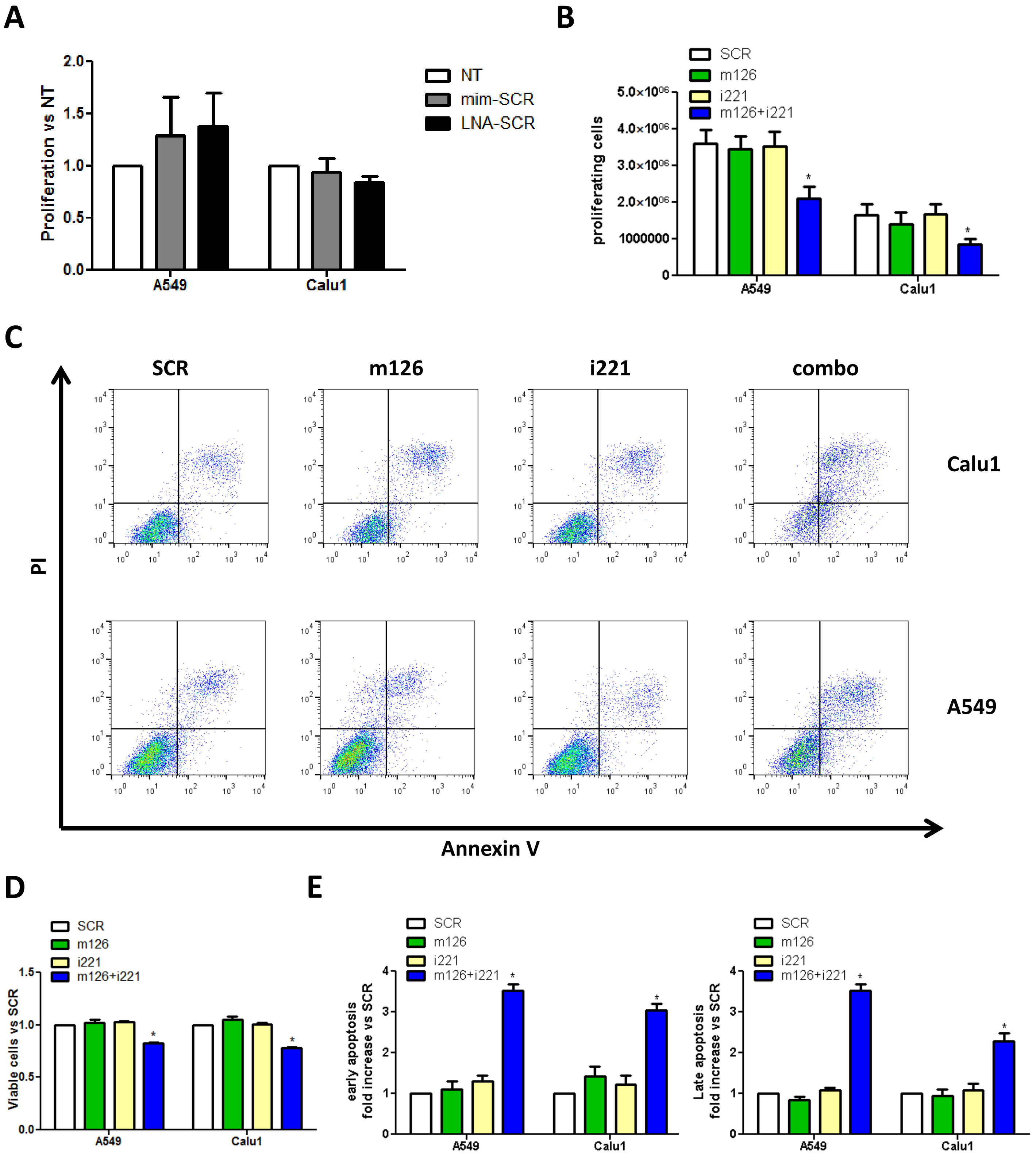
A
Proliferation vs NT
NT
mim-SCR
LNA-SCR
A549
Calu1
B
proliferating cells
SCR
m126
i221
m126+i221
A549
Calu1
C
SCR
m126
i221
combo
Calu1
A549
PI
Annexin V
D
Viable cells vs SCR
SCR
m126
i221
m126+i221
A549
Calu1
E
early apoptosis fold increase vs SCR
Late apoptosis fold increase vs SCR
SCR
m126
i221
m126+i221
A549
Calu1

Figure S2

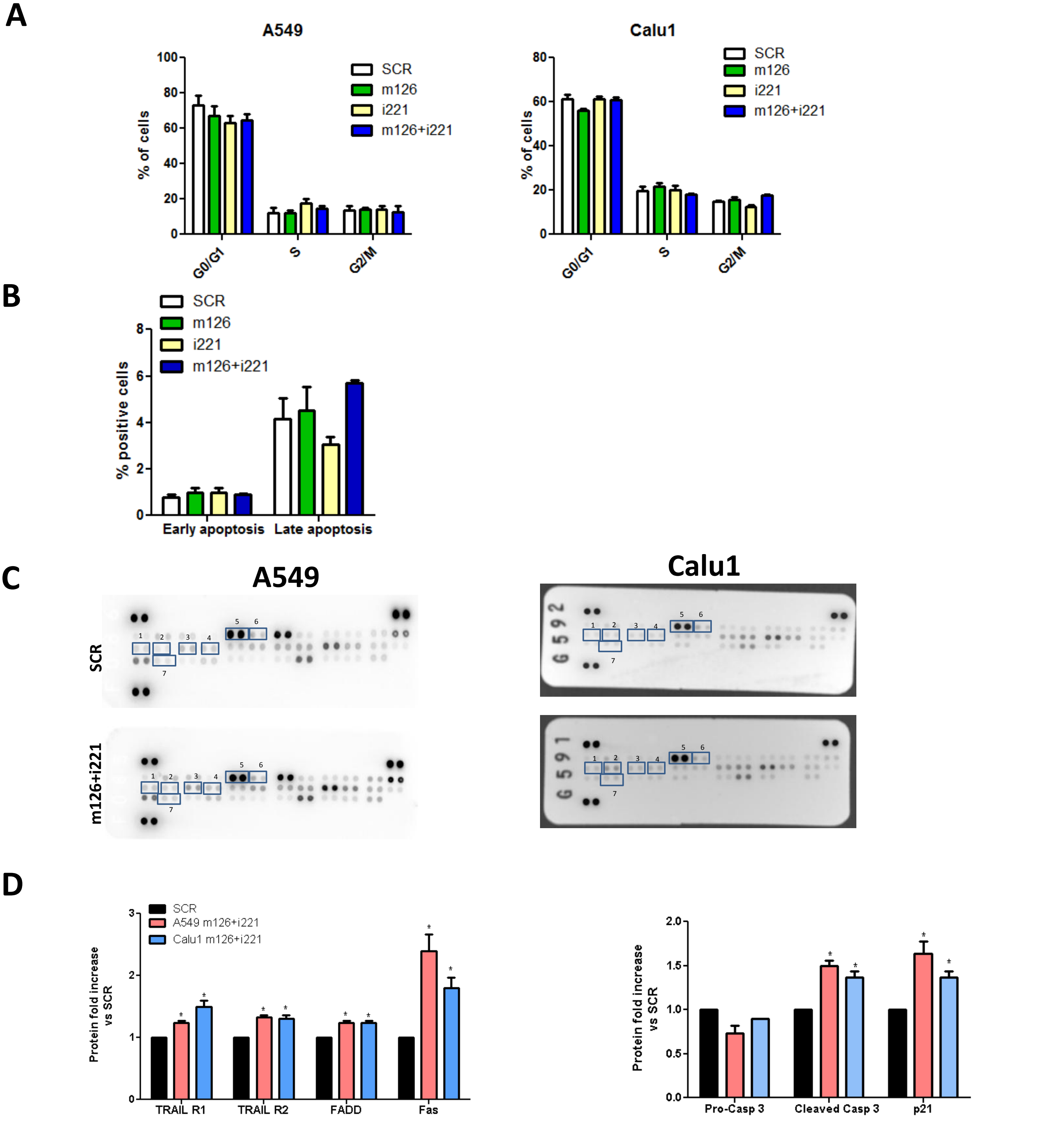
A
A549
100
80
60
40
20
0
% of cells
SCR
m126
i221
m126+i221
G0/G1
S
G2/M
Calu1
80
60
40
20
0
% of cells
SCR
m126
i221
m126+i221
G0/G1
S
G2/M
B
SCR
m126
i221
m126+i221
8
6
4
2
0
% positive cells
Early apoptosis
Late apoptosis
C
A549
SCR
m126+i221
1
2
3
4
5
6
7
Calu1
1
2
3
4
5
6
7
D
SCR
A549 m126+i221
Calu1 m126+i221
3
2
1
0
Protein fold increase vs SCR
TRAIL R1
TRAIL R2
FADD
Fas
2.0
1.5
1.0
0.5
0.0
Protein fold increase vs SCR
Pro-Casp 3
Cleaved Casp 3
p21

Figure S3

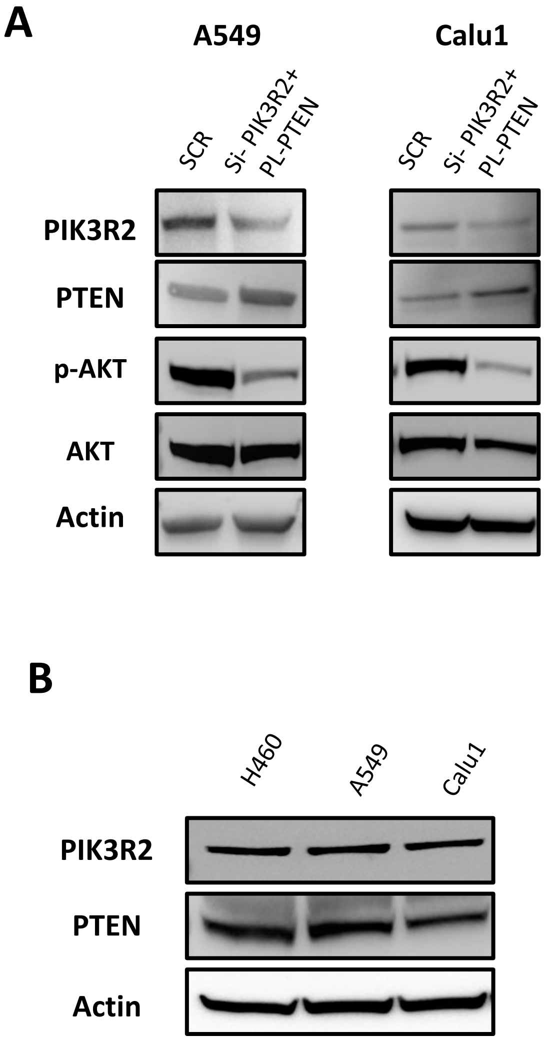
A
A549
Calu1
SCR
Si- PIK3R2+
PL-PTEN
SCR
Si- PIK3R2+
PL-PTEN
PIK3R2
PTEN
p-AKT
AKT
Actin
B
H460
A549
Calu1
PIK3R2
PTEN
Actin

Figure S4

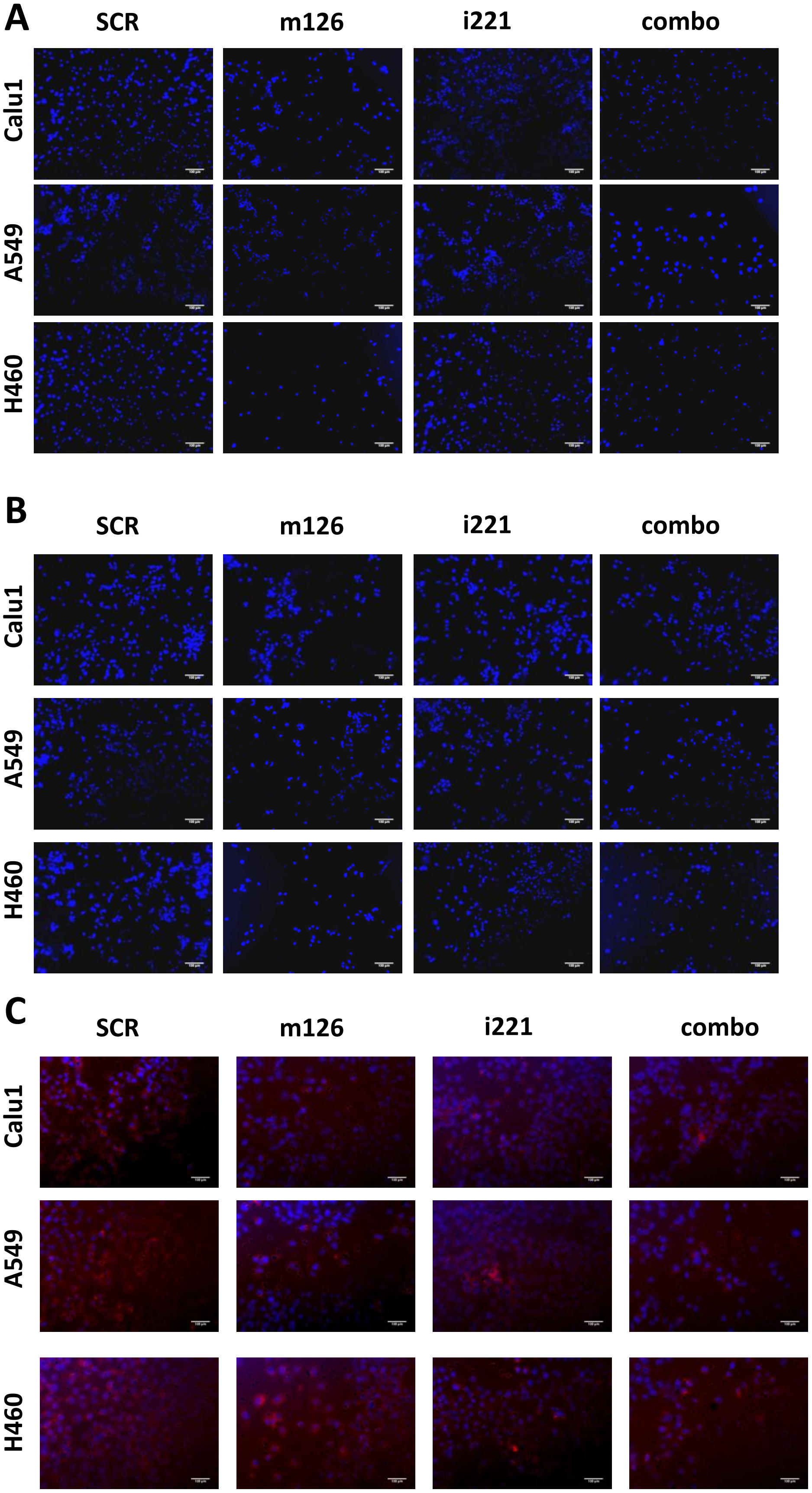
A
SCR
m126
i221
combo
Calu1
A549
H460
B
SCR
m126
i221
combo
Calu1
A549
H460
C
SCR
m126
i221
combo
Calu1
A549
H460

Figure S5

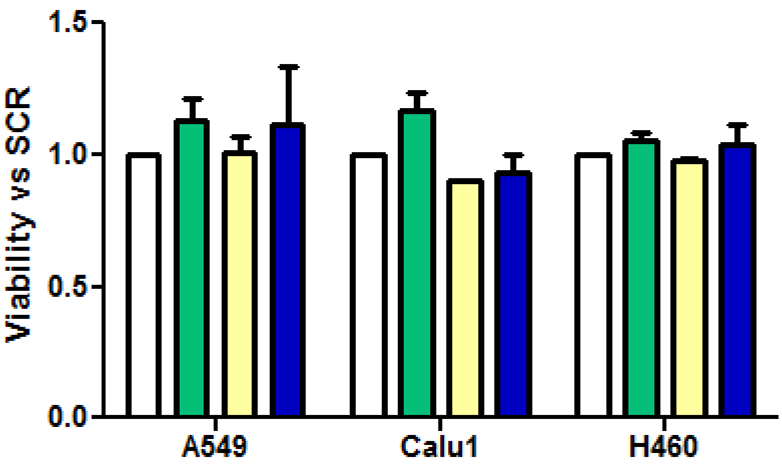


**Figure S6**

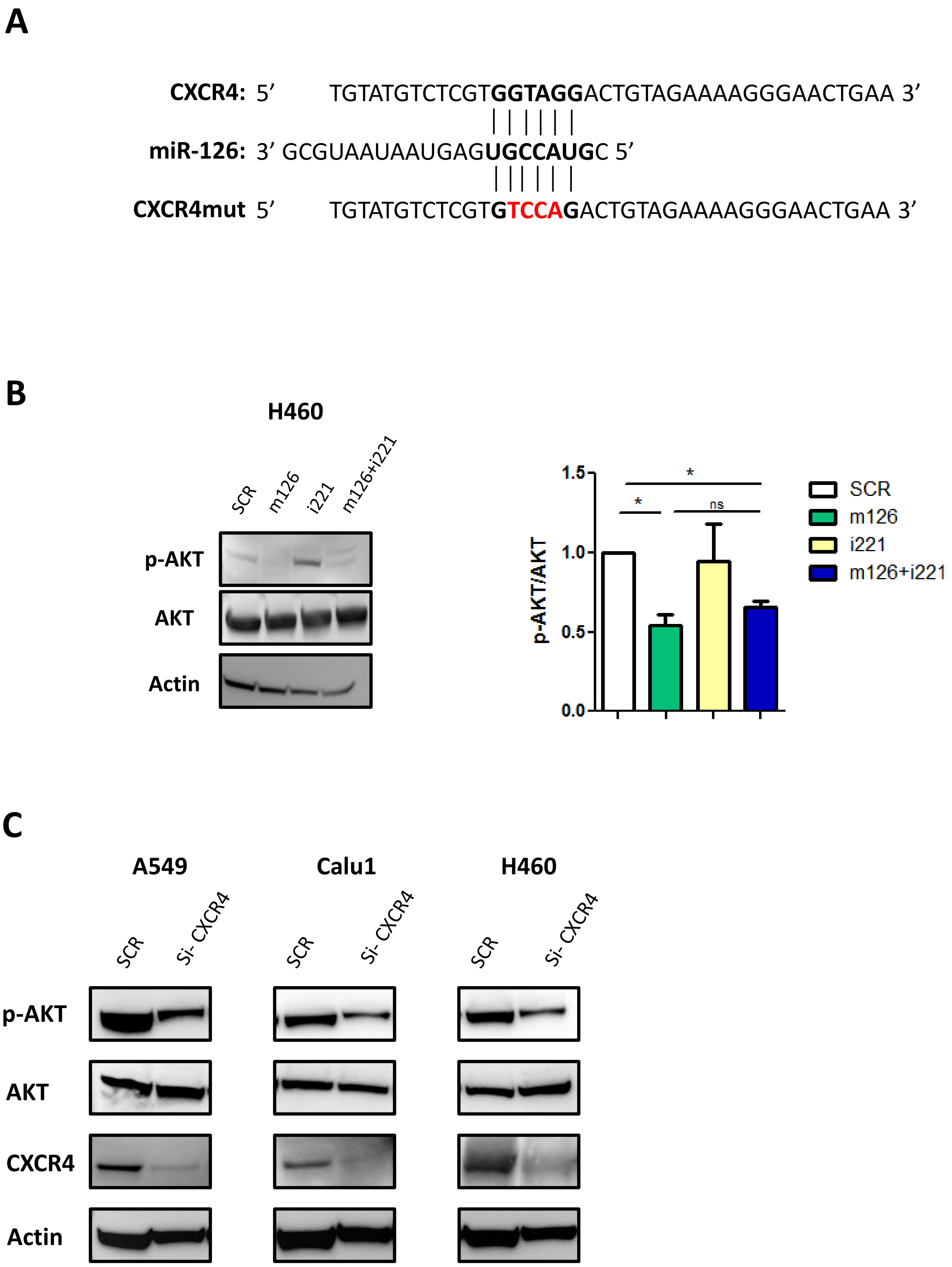

A
CXCR4: 5' TGTATGTCTCGTGGTAGGACTGTAGAAAGGGAACTGAA 3'
miR-126: 3' GCGUAAUAAUGAGUGCCAUGC 5'
CXCR4mut 5' TGTATGTCTCGTGTCCAGACTGTAGAAAGGGAACTGAA 3'
B
H460
SCR
m126
i221
m126+i221
p-AKT
AKT
Actin
p-AKT/AKT
1.5
1.0
0.5
0.0
*
*
ns
SCR
m126
i221
m126+i221
C
A549
Calu1
H460
SCR
Si- CXCR4
p-AKT
AKT
CXCR4
Actin


Figure S7

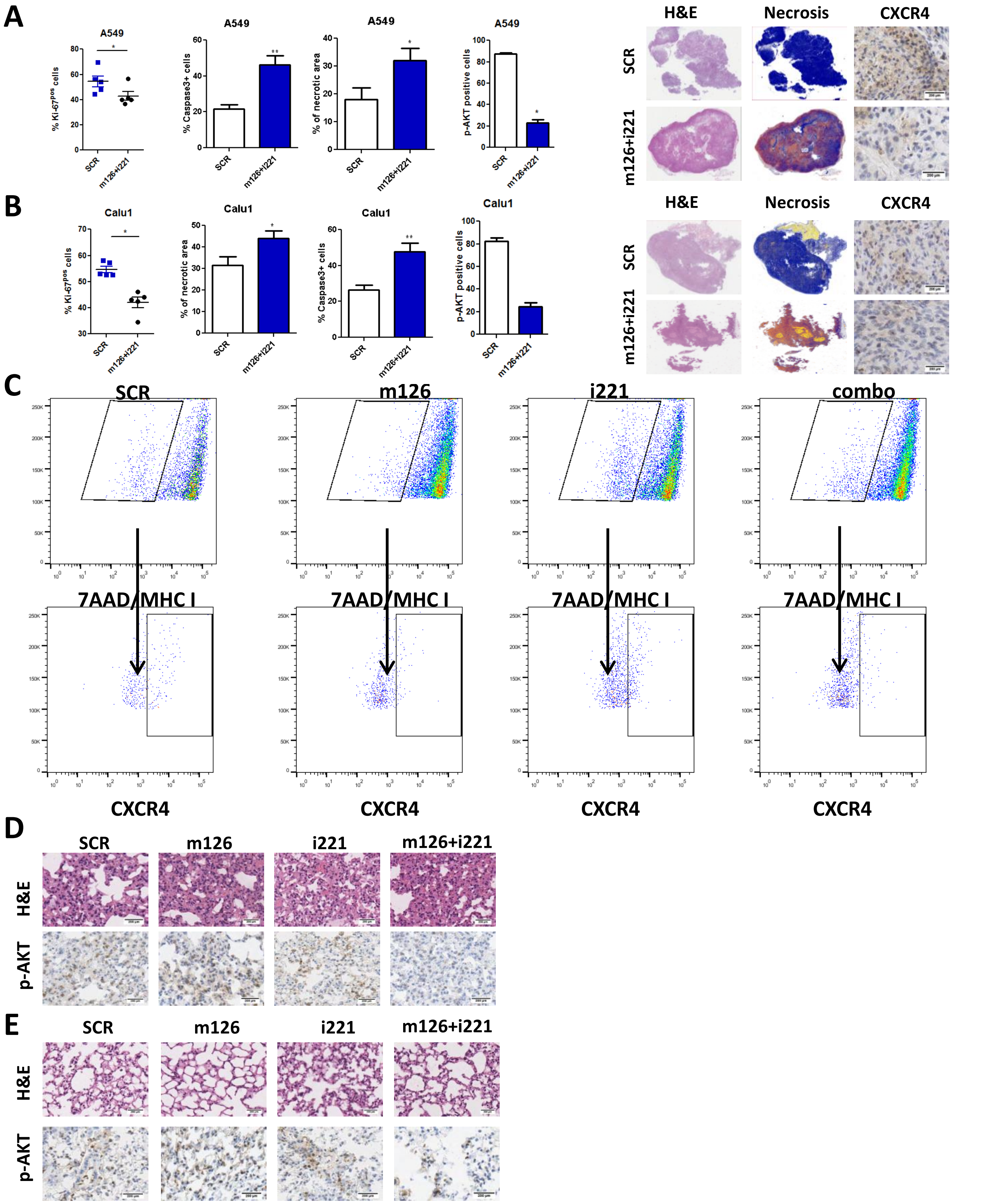

A
A549
% Ki-67pos cells
SCR
m126+i221
% Caspase3+ cells
% of necrotic area
p-AKT positive cells
H&E
Necrosis
CXCR4
B
Calu1
C
SCR
m126
i221
combo
7AAD/MHC I
CXCR4
D
p-AKT
E


Figure S8

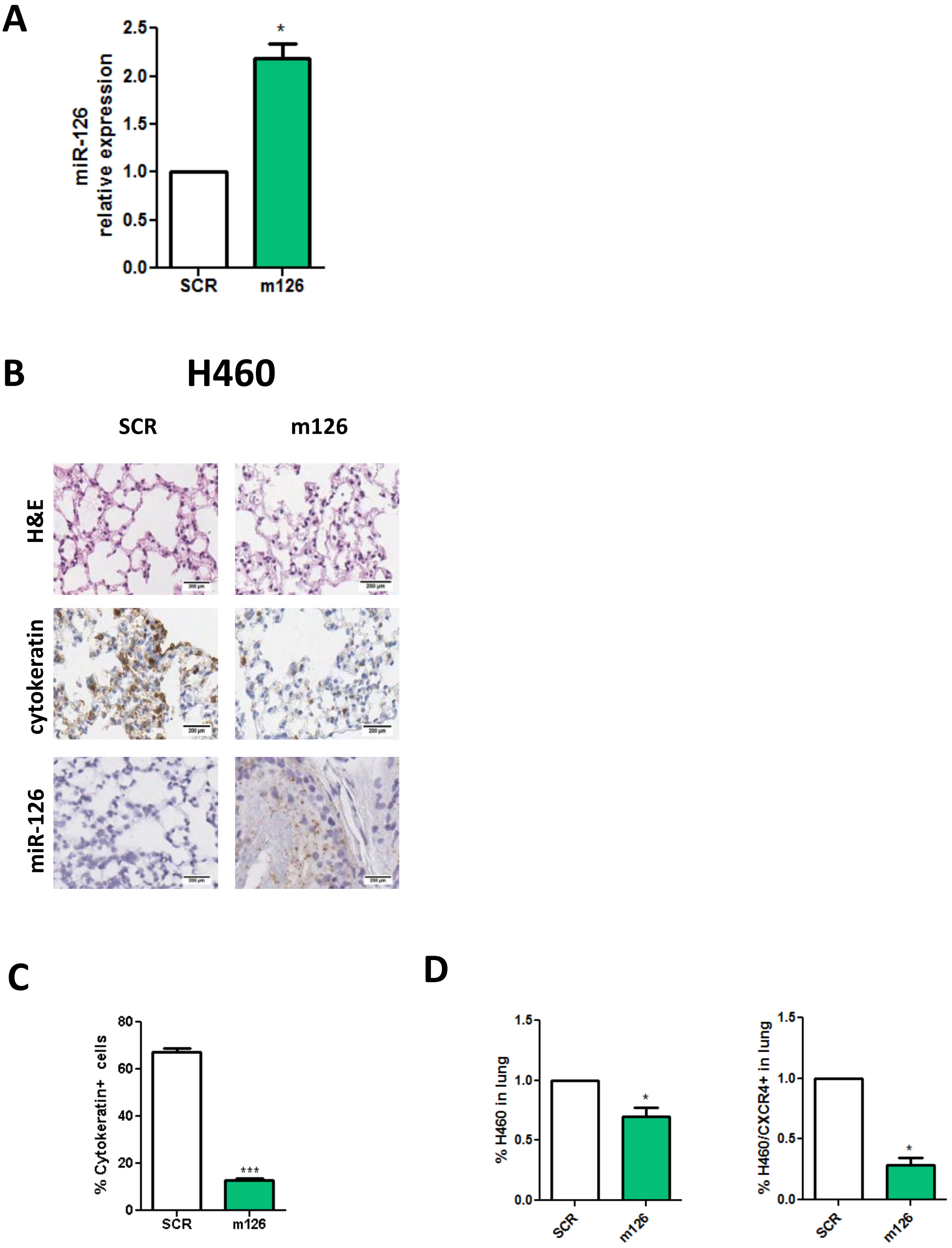
A
miR-126 relative expression
2.5
2.0
1.5
1.0
0.5
0.0
*
SCR
m126
B
H460
SCR
m126
H&E
cytokeratin
miR-126
C
% Cytokeratin+ cells
80
60
40
20
0
***
SCR
m126
D
% H460 in lung
1.5
1.0
0.5
0.0
*
SCR
m126
% H460/CXCR4+ in lung
1.5
1.0
0.5
0.0
*
SCR
m126

Figure S9

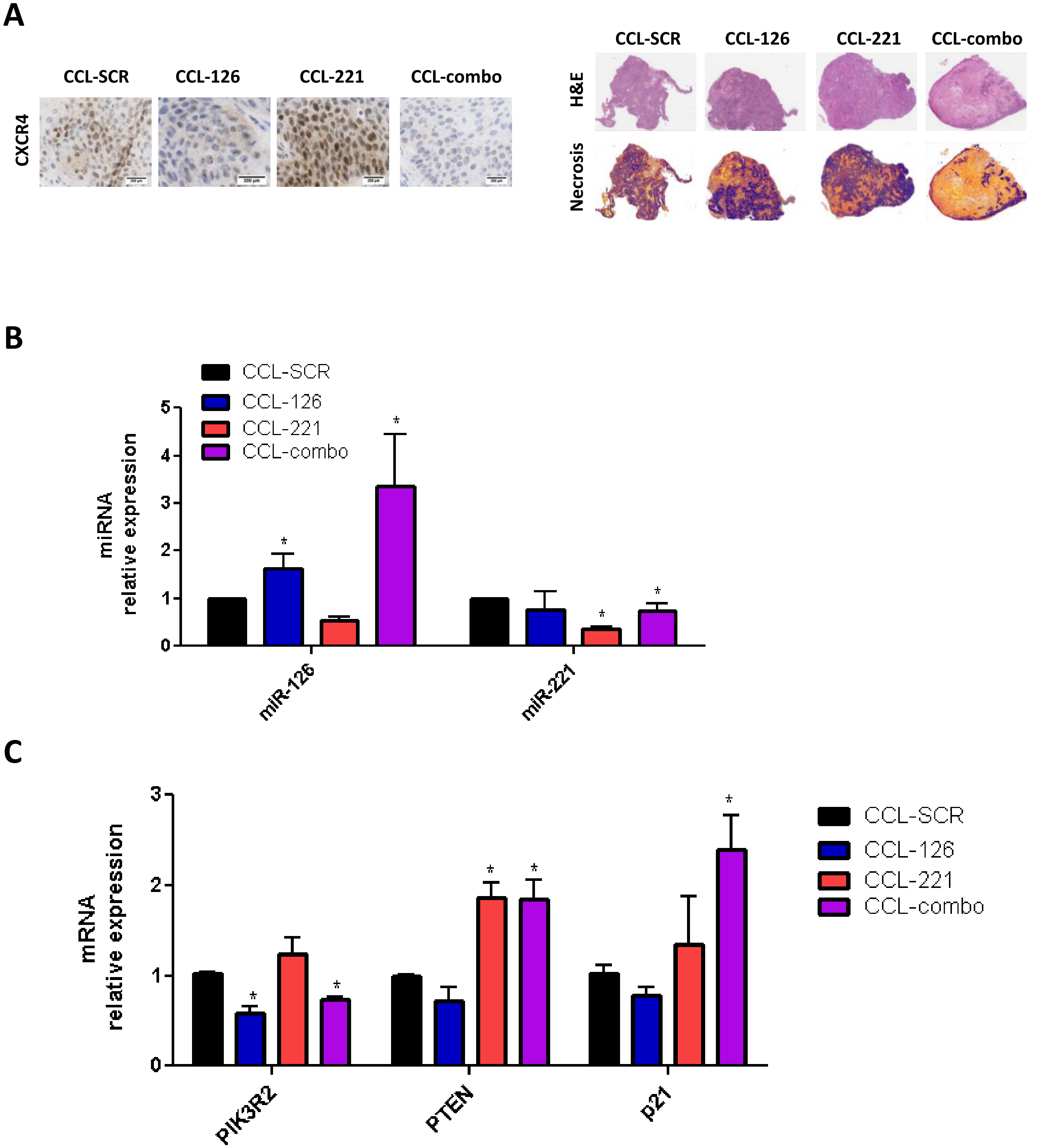
A
CCL-SCR
CCL-126
CCL-221
CCL-combo
CXCR4
H&E
Necrosis
B
CCL-SCR
CCL-126
CCL-221
CCL-combo
miRNA
relative expression
0
1
2
3
4
5
*
miR-126
miR-221
C
mRNA
relative expression
0
1
2
3
PIK3R2
PTEN
p21

Figure S10
