# Supplementary material for: Cotargeting of miR‐126‐3p and miR‐221‐3p inhibits PIK3R2 and PTEN, reducing lung cancer growth and metastasis by blocking AKT and CXCR4 signalling
Source: Mol Oncol. 2021 Jul 21;15(11):2969–88. doi: 10.1002/1878-0261.13036 (PMC8564655; doi:10.1002/1878-0261.13036)
Supplement: Supplementary file 2 — Table S1. List of detection probes. Table S2. List of miRNAs analyzed in lung cancer tissues. Table S3. Correlation analysis between de‐regulated miRNAs in lung cancer tissues. Table S4. Contingency table of lung cancer patients. Table S5. Contingency table of lung cancer patients. [file MOL2-15-2969-s002.docx]

**Co-targeting of miR-126-3p and miR-221-3p inhibits PIK3R2 and PTEN, reducing lung cancer growth and metastasis by blocking AKT and CXCR4 signaling**

*Daniela Di Paolo^1, § ,#^ Francesca Pontis^2,#^, Massimo Moro^2^, Giovanni Centonze^2,3^, Giulia Bertolini^2^, Massimo Milione^3^, Mavis Mensah^2^, Miriam Segale^2^, Ilaria Petraroia^2^, Cristina Borzi^2^, Paola Suatoni^4^, Chiara Brignole^1^, Patrizia Perri^1^, Mirco Ponzoni^1^, Ugo Pastorino^4^, Gabriella Sozzi^2, ‡^ and Orazio Fortunato^2,*,‡^*

Suppl. Table 1. List of Detection Probes

| Probe | RNA Tm (°C) | T hyb (°C) | Probe sequence |
| --- | --- | --- | --- |
| Hsa-miR-21 | 83 | 53 | TCAACATCAGTCTGATAAGCTA |
| Hsa-miR-126 | 84 | 54 | GCATTATTACTCACGGTACGA |
| Hsa-miR-221 | 84 | 54 | GAAACCCAGCAGACAATGTAGCT |
| Hsa-miR-451 | 82 | 52 | AACTCAGTAATGGTAACGGTTT |
| Hsa-miR-486 | 92 | 62 | CTCGGGGCAGCTCAGTACAGGA |
| Hsa-miR-210 | 84 | 54 | CUGUGCGUGUGACAGCGGCUGA |
| Hsa-miR-30a | 84 | 54 | UGUAAACAUCCUCGACUGGAAG |
| Scramble | 87 | 57 | GTGTAACACGTCTATACGCCCA |

Tm: melting temperature; T hyb: hybridization temperature.

**Suppl. Table 2. List of miRNAs analyzed in lung cancer tissues**

| **MiRNAs** | |
| --- | --- |
| let7-c | miR-205 |
| miR-1 | miR-210 |
| miR-7-3p | miR-219 |
| miR-15a | miR-221 |
| miR-16 | miR-222 |
| miR-21 | miR-324-5p |
| miR-29b-5p | miR-339-5p |
| miR-30a | miR-340 |
| miR-30d-3p | miR-346 |
| miR-30e | miR-369-3p |
| miR-34b | miR-429 |
| miR-126-3p | miR-451 |
| miR-126-5p | miR-486-5p |
| miR-128a | miR-518a-3p |
| miR-144 | miR-518e-3p |
| miR-193a-5p | PremiR-128a |
| miR-200b | PremiR-185 |

**Suppl.Table 3. Correlation analysis between de-regulated miRNAs in lung cancer tissues**

|  | **miR-221** | | **miR-222** | | **miR-21** | | **miR-210** | |
| --- | --- | --- | --- | --- | --- | --- | --- | --- |
|  | **Spearman r** | **p value** | **Spearman r** | **p value** | **Spearman r** | **p value** | **Spearman r** | **p value** |
| **miR-30a** | -0,129 | 0,453 | 0,818 | <0,0001 | 0,208 | 0,225 | 0,524 | 0,001 |
| **miR-126** | **-0,756** | **<0,0001** | 0,089 | 0,607 | 0,073 | 0,671 | -0,065 | 0,706 |
| **miR-451** | 0,466 | 0,006 | -0,295 | 0,09 | **-0,479** | **0,004** | -0,255 | 0,146 |
| **miR-486** | -0,105 | 0,544 | 0,616 | <0,0001 | 0,690 | <0,0001 | 0,522 | 0,001 |

**Suppl. Table 4. Contingency table of lung cancer patients**

|  | **miR-221 low** | **miR-221 high** |
| --- | --- | --- |
| **miR-126 low** | 2 | 17 |
| **miR-126 high** | 17 | 2 |

**Suppl. Table 5. Contingency table of lung cancer patients**

|  | **miR-21 low** | **miR-21 high** |
| --- | --- | --- |
| **miR-451low** | 7 | 12 |
| **miR-451 high** | 12 | 7 |

**FIGURE LEGENDS**

**Figure S1. miRNAs de-regulation in lung cancer. A)** Quantification of miRNAs expression in normal and tumor tissues (n=4). **B)** Histograms showing 6 miRNAs levels in normal, non tumorigenic and tumor lung epithelial cells (n=3). **C)** Graphs show miR-126, miR-221 reciprocal levels in different cancer cell lines (n=3)**. D)** Cells were transfected with miR-126 mimic (m126) and LNA-221 (i221) and miRNA levels were measured at 72h by Real Time PCR (n=3). Data are expressed as mean ± S.E.M

**Figure S2. Proliferation and apoptosis analysis. A)** Proliferation of lung cancer cells transfected with negative controls such as mimic-SCR or inhibitor SCR (LNA-SCR) (n=5). **B)** Number of proliferation cells after miR-126 replacement and miR-221 inhibition after 120 h post transfection (n=5). **C)** Representative images of apoptotic analysis by Flow Cytometry. **D)** Fold increase of viable transfected cells compared to SCR control (n=5) **E)** Apoptotic rate in A549 and Calu1 after 120h post transfection (n=5). Data are expressed as mean±S.E.M

**Figure S3. Apoptosis analysis A)** Graphs show relative percentage of cell cycle phases after miRNA transfection. **B)** Apoptotic rate graphs of human non tumorigenic epithelial cell lines transfected with miR-126 mimics and miR-221 inhibitors compared to SCR control (50 nM, n = 5 for each assay). **C)** Representative images of 1-TRAILR1; 2-TRAILR2; 3-FADD; 4-Fas/TNFRSF6; 5-Pro-Caspase 3; 6-Cleaved Caspase3; 7-p21. **D**) Histogram of protein array quantification showing TRAIL activation after m126+i221 transfection (n=3 for each cell lines). Activation of apoptosis was confirmed by cleavage of Caspase 3 and p21 increase. Data are expressed as mean±S.E.M

**Figure.S4. PIK3R2 and PTEN are miR-126 and miR-221 targets. A)** Western blot bands confirmed PIK3R2 inhibition after silencing and PTEN over-expression after plasmid transfection (n=3) in Calu1 and A549. pAKT activation was evaluated by WB in si-PIK3R2 + PL-PTEN treated cells. **B)** PIK3R2 and PTEN expression in all the three lung cancer cell lines.

**Figure. S5. Migratory and invasive capacity of lung cancer cells.** Representative images of A549, Calu1 and H460 transfected with miR-126 mimics and miR-221 inhibitors and analyzed by migration (**A**), invasion (**B)** and trans-endothelial migration (**C**) assays.

**Figure. S6. Proliferation analysis after 24 h post transfection.**  Bar plots illustrate lung cancer cells viability rate at 24h after miR-126-3p replacement and miR-221-3p inhibition compared to SCR control (n=5 for each cells)

**Figure. S7. CXCR4 is fundamental for metastatic dissemination. A)** Putative binding sequence on CXCR4 3’UTR for miR-126. In red the mutated sequence for CXCR4. **B**) Representative images and quantification of AKT phosphorylation in H460 after miRNA modulation (n=3). **C)** Representative WB images of AKT phosphorylation in lung cancer cells after CXCR4 modulation by siRNA (n=3 for each cell lines).

**Figure. S8. In vivo assays. A)** Quantification of Ki-67, Caspase 3, necrotic cells and CXCR4 in A549 (**A**) or Calu1 (**B**) after miRNA modulation. Blue staining revealed the nucleus of the cells. **C)** Gating strategy for the analysis of metastatic dissemination in the lungs**. D**) Images of H&E and p-AKT in A549 injected in the lung of mice. Scale bar: 200µm **E**) Representative images of H&E and phospho-AKT staining of the lungs from H460 transfected with miR-126-3p and control.

**Figure. S9. miR-126 replacement using nebulized aerosol inhalation. A)** Graphs show miR-126 in treated mice compared to controls (n=3). **B**) Representative images and quantification of H&E and pan-cytokeratin staining of the lungs from H460 treated with nebulized miR-126-3p mimics. Scale bar: 200µm. **C**) Number of cytokeratin positive cells in the lung after miR-126 nebulization **D**) Flow cytometric quantification of H460-disseminating cells and H460-CXCR4 positive in the lung of treated mice. Data are expressed as mean ± S.E.M

**Figure. S10. PDX was treated with lipid-nanoparticles. A)** CXCR4 and necrosis IHC images of PDXs treated with lipid-nanoparticles. Blue staining revealed the nucleus of the cells**.** Scale bar: 200µm **B)** Graphs show miR-126 and miR-221 in treated mice compared to controls (n=3). **B)** Real Time PCR graphs of lung cancer PDX treated with lipid nanoparticles. Data are expressed as mean ± S.E.M
